# Direct Action of Non-Digestible Oligosaccharides against a Leaky Gut

**DOI:** 10.3390/nu14214699

**Published:** 2022-11-07

**Authors:** Maria Eleni Mavrogeni, Mostafa Asadpoor, Paul A. J. Henricks, Ali Keshavarzian, Gert Folkerts, Saskia Braber

**Affiliations:** 1 Division of Pharmacology, Utrecht Institute for Pharmaceutical Sciences, Faculty of Science, Utrecht University, 3584 CG Utrecht, The Netherlands; 2 Division of Gastroenterology, Department of Internal Medicine, Rush University Medical Center, Chicago, IL 60612, USA

**Keywords:** non-digestible oligosaccharides, intestinal epithelial barrier, tight junctions, paracellular permeability, TEER, leaky gut, commensal microbiota

## Abstract

The epithelial monolayer is the primary determinant of mucosal barrier function, and tight junction (TJ) complexes seal the paracellular space between the adjacent epithelial cells and represent the main “gate-keepers” of the paracellular route. Impaired TJ functionality results in increased permeation of the “pro-inflammatory” luminal contents to the circulation that induces local and systemic inflammatory and immune responses, ultimately triggering and/or perpetuating (chronic) systemic inflammatory disorders. Increased gut leakiness is associated with intestinal and systemic disease states such as inflammatory bowel disease and neurodegenerative diseases such as Parkinson’s disease. Modulation of TJ dynamics is an appealing strategy aiming at inflammatory conditions associated with compromised intestinal epithelial function. Recently there has been a growing interest in nutraceuticals, particularly in non-digestible oligosaccharides (NDOs). NDOs confer innumerable health benefits via microbiome-shaping and gut microbiota-related immune responses, including enhancement of epithelial barrier integrity. Emerging evidence supports that NDOs also exert health-beneficial effects on microbiota independently via direct interactions with intestinal epithelial and immune cells. Among these valuable features, NDOs promote barrier function by directly regulating TJs via AMPK-, PKC-, MAPK-, and TLR-associated pathways. This review provides a comprehensive overview of the epithelial barrier-protective effects of different NDOs with a special focus on their microbiota-independent modulation of TJs.

## 1. Introduction

A functional intestinal barrier is indispensable for maintaining gut and systemic homeostasis, separating the external environment and the strictly regulated internal milieu and absorbing nutrients and fluids while preventing the invasion of pathogens/pathobionts and other noxious molecules, such as pro-inflammatory bacterial products such as endotoxins, environmental toxins, and antigens [1,2]. Conservation of a homeostatic gut barrier requires fine-tuned operations of its components, i.e., from the luminal to the basolateral surface: the gut microbiota, the mucus layer, the epithelial monolayer, and immune cells of the lamina propria [3,4]. The biliary juices, gastric, and pancreatic acids found in the lumen, defend against pathogens and other antigenic factors. Gut flora present in the lumen exerts multiple beneficial activities on the host. First, commensal bacteria halt the colonization of pathogens by (1) the production of antimicrobial peptides named bacteriocins, (2) competition for nutrients essential for the growth of pathogens, and (3) pH modification of the luminal contents [1,5]. In addition, interactions between gut microbes and epithelial cells have been established as key contributors to the modulation of epithelial permeability via cell–cell junction formation and reinforcement of the mucus layer [3,6,7]. The second defense mechanism is the microclimate, i.e., the outer and inner mucus layers and the unstirred water layer called glycocalyx, located above the epithelium. The mucus-like glycocalyx averts bacterial adhesion physically. Goblet cells of the epithelium produce and secrete mucin, a heavily glycosylated protein forming the mucus layer. This layer facilitates the safe habitancy of commensals close to the epithelium and contains secretory immunoglobulin A (sIgA) derived from lamina propria plasma cells. sIgA is the principal antibody present in mucosal secretions, involved in eliminating invading pathogens via their entrapment in the mucus layer and maintaining balanced commensal populations [1,5]. However, the mucus layer does not confer a substantial barrier to transmucosal water and solute flux, as this is the responsibility of the intestinal epithelial layer [8]. The epithelium is a layer of polarized columnar epithelial cells that separates the underlying lamina propria from the intestinal luminal environment and comprises several cell types, including enterocytes and enteroendocrine Paneth and goblet cells [9]. Enterocytes mediate the absorption and transport of luminal contents into the internal body compartments and hinder injurious factors by chloride secretion. Paneth cells are involved in the production of antimicrobial peptides (AMPs), which combat pathogenic stimuli [1,5]. Cell–cell junctional complexes between the intestinal epithelial cells (IECs) form a tight barrier that strictly regulates paracellular permeability and is pivotal for the integrity of the gut barrier [3,9]. Thus, the epithelial monolayer is the primary determinant of mucosal barrier function [8]. The fourth level of defense is the lamina propria, the layer beneath the epithelial cells, which comprises immunoglobulin- and cytokine-secreting innate and adaptive immune cells, such as macrophages, mast cells, and T-regulatory cells. These cells cooperate to confront and eliminate invaders through well-orchestrated anti-inflammatory responses, influence epithelial permeability and interact with the endocrine and enteric nervous systems. The enteric nervous system activates endocrine and secretomotor mechanisms resulting in intestinal propulsive motility, constituting the final component of the intestinal barrier [1,5].

The maintenance of a homeostatic intestinal epithelial barrier is of paramount importance. Disturbed barrier function is directly linked to increased paracellular and transcellular intestinal permeability, a hallmark of increased invasion of pathogens and permeation of pro-inflammatory luminal factors into the systemic circulation with subsequent systemic inflammation and initiation of numerous diseases [10]. The so-called “leaky gut syndrome” has been associated with several chronic inflammatory intestinal diseases and conditions, including inflammatory bowel disease (IBD), irritable bowel syndrome (IBS), and celiac disease, but also with numerous extraintestinal/systemic diseases and disorders, namely obesity, types 1 and 2 of diabetes, Alzheimer’s disease, Parkinson’s disease, major depression disorder, and autism spectrum disorders [1,11]. Moreover, increased epithelial permeability may occur with aging and physiological conditions such as stress or intensive exercise without the presence of predisposing conditions [9].

Over recent years, there has been an increasing interest in factors capable of reinforcing the intestinal epithelial barrier via their barrier-protective or -reparatory properties. Given the well-established association between gut dysbacteriosis and impaired barrier function, an already widely applied therapeutic approach involves the restoration of imbalanced gut microbiota populations [12]. Manipulation of commensal microbiota via bacteria-based therapy restores dysbiotic populations, alleviates inflammation, and preserves healthy ones [13,14]. Bacteriotherapy is now a well-established strategy achieved with traditional probiotic or next-generation probiotic supplementation or fecal microbiota transplantation [15,16]. The term probiotics refers to “live microorganisms that, when administered in adequate amounts, confer a health benefit to the host” [16]. It is now well-recognized that commensals and probiotics reinforce the epithelial tight junctions (TJs), i.e., the most pivotal cell–cell contacts, via both their metabolites and bacterial cell structures [3,7,12,17].

Another strategy to manipulate the microbiota community and restore dysbiotic populations is the use of prebiotics. The term prebiotics refers to “poorly absorbed carbohydrates that, when administered in adequate amounts, promote the growth of bacteria capable of conferring a health benefit to the host” [18]. Indeed, prebiotic supplementation has been investigated as an indirect strategy for enhanced barrier function, considering that prebiotic agents promote the growth of health-beneficial commensals. Apart from their microbe-related beneficial activity, emerging evidence demonstrates that prebiotics have direct barrier-protective properties, i.e., via mechanisms independent of the gut flora, including direct induction of TJ signaling [12,19,20]. Hence, an interesting approach aiming at protecting or even repairing the intestinal epithelial barrier would be the use of nutraceuticals, such as prebiotics, with barrier-enhancing capabilities through their direct and indirect promotion of TJ functionality. Non-digestible oligosaccharides (NDOs) are prebiotic fibers that have gained tremendous attention due to their multiple health-beneficial properties [20,21]. NDOs are complex carbohydrates usually consisting of 3 to 10 sugar moieties. Once ingested, NDOs resist hydrolysis by salivary and digestive enzymes and reach the large intestine intact, where they are finally hydrolyzed by enzymes produced by the colonic bacteria [21]. Human milk oligosaccharides (HMOs) present in human breast milk are the first prebiotics encountered in life, promoting the establishment of balanced microbiota populations and healthy mucosal immunity [22]. Fructooligosaccharides (FOS) and galactooligosaccharides (GOS) are well-established and exhaustively studied typical prebiotics that, due to their structural and functional resemblance to HMOs, are extensively added in infant formulas [23]. Numerous other categories have been identified, including the alginate-oligosaccharides (AOS), chitin/chitosan-oligosaccharides (COS), mannan-oligosaccharides (MOS), xylooligosaccharides (XOS), pectic-oligosaccharides (POS), soybean oligosaccharides (SOS), and isomaltooligosaccharides (IMOS) [24]. 

In this review, the main point of interest is the intestinal epithelial TJs and their regulation by prebiotic fibers, with a special focus on the widely used FOS and GOS and the recently emerged AOS and COS. Furthermore, the available data for MOS and XOS are presented. To better understand the underlying mechanisms implicated in the modulation of paracellular permeability by these NDOs, the most prominent signaling pathways responsible for the strict regulation of TJ abundance, localization, and dynamics are initially discussed. Moreover, the roles of two principal factors affecting epithelial permeability via TJs’ (patho)physiological regulation are analyzed, i.e., intestinal inflammation and the gut microbiota [7]. Having comprehended the principal factors that govern the intestinal epithelial paracellular permeability, the effects of the NDOs on the TJ complex will be summarized based on in vitro and in vivo studies to, as far as possible, connect these observations, with a particular emphasis on the microbiota-independent effects of the NDOs on intestinal TJs.

## 2. Sealing the Paracellular Route of the Intestinal Epithelial Barrier

### 2.1. Junctional Network of the Intestinal Epithelial Layer

**Figure 1:**
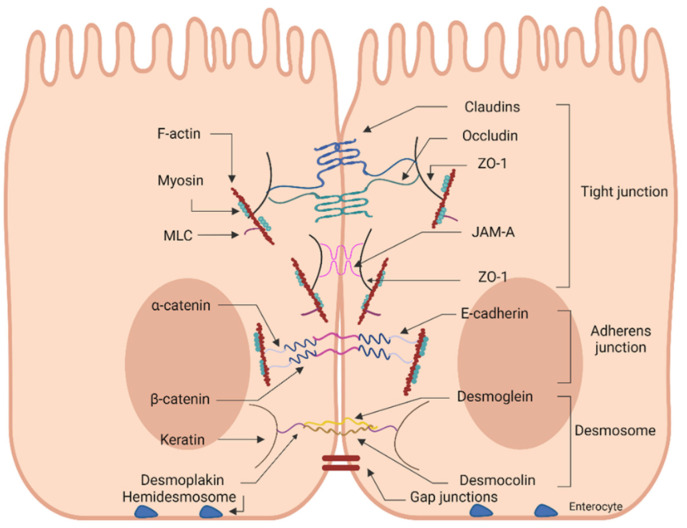
Junctional complex of IECs from apical to basal: TJs (ZO-1, -2, -3, occludin, claudins, JAM-A/1, -B/2, -C/3) serve as the principal paracellular barrier. AJs (cadherin-1, -2, α-, β-, γ-, δ-catenins) form the adhesion belt and hold together adjacent cells. TJs and AJs interact with F-actin to form the apical junctional complex. Desmosomes (desmoglein, desmocollin, desmoplakin, keratin) are “buttonlike” points of intercellular contact fixing cells together. Gap junctions (connexins) serve in cell–cell communication. Created with BioRender.com. AJ, Adherens junctions; IECS, Intestinal epithelial cells; TJ, Tight junctions.

Solutes pass across the intestinal epithelium either through the cells, i.e., transcellularly, or between the cells, i.e., paracellularly, depending on the solute properties. Small hydrophilic and lipophilic molecules, mainly nutrients, pass through the transcellular route, while larger ones, such as proteins, are internalized via the paracellular route [1,2]. The passage between adjacent cells is well-guarded by junctional complexes that join each unit of epithelial cells [9]. The epithelial junctional complex is comprised of four components: tight junctions (TJs), adherens junctions (AJs), desmosomes, and gap junctions (GJs) [1,25] (Figure 1). Each TJ is formed by the assembly of various proteins, resulting in a multifunctional complex located near the apical side of the lateral membrane of the epithelium [26,27]. The TJ assembly is the principal determinant of the intestinal physical barrier integrity, regulating the permeability of the paracellular route [27]. Beneath the TJs, the adherens junctions (AJs), also named intermediate junctions or “belt desmosome”, are positioned [4,9]. AJs are composed of proteins belonging to the cadherin family (E-cadherins) and the α-, β-, γ- and δ-catenins, and are involved in cell–cell adhesion and intracellular signaling [1,9]. Due to the apical location and direct association of TJs and AJs to the cytoskeleton via the perijunctional filamentous actin (F-actin) ring, their complex is called the apical junctional complex [1]. Below this complex, at the basolateral interspace of the epithelial cells, the desmosomes, or maculae adhaerentes/adherens, are located and can also be considered part of the apical junctional complex [4,8]. These adhesive structures are formed by interactions between desmoglein, desmocollin (also members of the cadherin family), and the connecting proteins desmoplakin and keratin and are involved in cell–cell adhesion [1,4,9]. Ultimately, the gap junctions operate as intercellular channels for ions and small molecules between adjacent cells and thus are involved in intercellular communication [1]. Interestingly, the cell junctions of the gastrointestinal (GI) tract are probably the most complex compared to the ones found on the endothelium and epithelium of other organs, with apparent variations along both the length and within the crypt-villi axes [28].

### 2.2. Importance of TJ Network

Even though all the junctional complex components are crucial for preserving a functional barrier via their adhesive and mechanical properties, only TJs represent the “gate-keepers” of the paracellular route [29]. TJs seal the space between neighboring cells, forming a continuous intercellular barrier across the interspace of epithelial cells [20,30]. This barrier is only permeable via paracellular diffusion to medium-sized hydrophilic molecules (≤600 Da in vivo and ≤10 kDa in vitro) and subsequently, under normal conditions, is impermeable to antigenic macromolecules, e.g., microorganisms, while ions and positively charged molecules pass more effortlessly [1]. Having mentioned the above, it is clear that the paracellular transport of ions, water, nutrients, and pathogens is under the stringent regulation of TJs. In addition, TJs mediate other essential functions, such as establishing cell polarity (fence function) and regulating epithelial cell proliferation and differentiation [31]. In general, there are three distinct paracellular permeability pathways recognized. The “leak” and “pore” pathways determine intestinal permeability as dictated by the strict regulation of TJs present in intact epithelia [5,8]. The pore pathway is a high-capacity, size- and charge-selective route, while the leak pathway is a low-capacity route with limited selectivity [8]. The “unrestricted flux” pathway is the principal route across ulcerated and corroded epithelia. This pathway is dominant during apoptotic conditions due to pathological states and permits the passage of luminal antigens to the lamina propria [5,8]. The unrestricted pathway is high-capacity and lacks size and charge selectivity. Consequently, high molecular weight (MW) proteins and whole microorganisms can freely cross it [8]. TJs are not just static barriers but highly dynamic structures that are continually being restructured upon the presence of external stimuli such as commensal microbes, pathogens, and nutrient residues. Thus, the TJ complex is one of the most critical components of the intestinal barrier, not only regulating the flux of nutrients, ions, and water but also limiting the entry of pathogens [9]. As mentioned above, abrogation or injury of the intestinal barrier due to a failure to form or conserve the epithelial TJs results in infections, increased systemic inflammation due to bacterial-lipopolysaccharide (LPS) absorption, and induction of numerous intestinal and extra-intestinal diseases and disorders [10].

## 3. Tight Junctions: Components, Regulation, and Gut Flora

### 3.1. Basic Components of the TJ Complex

As discussed above, TJs are regarded as the rate-limiting factor for paracellular permeability and regulate the paracellular transport of molecules in a size- and charge-dependent manner in response to numerous stimuli. Nonetheless, the precise role of each protein involved in the TJ network (more than 40 proteins) has not yet been fully determined [32]. Each TJ is a multiprotein complex composed of integral transmembrane proteins extending into the paracellular spaces between adjacent cells and cytosolic scaffold proteins connected to cell cytoskeleton structures [32] (Figure 1). TJ strands can be formed between two or three adjacent cells, named bicellular or tricellular TJs, respectively [27]. The transmembrane proteins include the MARVEL domain proteins occludin, tricellulin, and marvelD3 (all belong to the TJ-associated MARVEL proteins; TAMPS), claudins, and the TJ-associated junctional adhesion molecules (JAM-1, -2 and -3) [1,33]. The extracellular domains of the transmembrane proteins connect to adjacent cells, forming networks of linking strands [31]. The attachment of this complex to the cytoskeleton of the neighboring cells occurs via the intracellular scaffolding or plaque proteins zonula occludens-1, -2, -3 (ZO-1, ZO-2, ZO-3) and peripheral adaptor proteins, namely symplekin and cingulin [1]. The intracellular domains of the transmembrane proteins are connected to the cytosolic scaffold proteins, kinases, and cytoplasmic filaments [31]. The cell cytoskeleton structures involve the microtubules and micro- and intermediate filaments that extend throughout the cytosol [4]. Maintenance of this structure is crucial for the cell itself and the conservation of barrier integrity. The transmembrane proteins appear embedded in the cell membrane and are either tetra-span (claudins, occludin, tricellulin) or single-span (JAMs). In the first case, the proteins are formed by four transmembrane domains and two extracellular loops, with the N- and C-terminals located in the cytoplasm [9]. Interactions between extracellular domains of the transmembrane proteins of neighboring cells regulate the paracellular passage of molecules by sealing the paracellular space [27]. Below, the most significant components of the TJ protein complex are briefly discussed.

#### 3.1.1. Claudins

In humans, at least 27 gene members of the claudin family have been found [3,29]. Claudins are responsible for forming the actual paracellular pore within the TJ assembly and regulation of the paracellular space and thus constitute one of the primary regulators of TJ permselectivity [28,30,32]. Claudin-TJ strands are formed by four transmembrane passages that produce two extracellular loops, which interact with claudins of the same (homo-interactions) or adjacent cells (hetero-interactions) via both cis- and trans-interactions [28]. The intracellular domains of claudins are connected to the plaque proteins, which offer a structural scaffold to the TJs [27]. The expression of TJ proteins in the GI tract is tissue- and age-dependent but also varies in the presence of disease [29,33]. In the intestine, claudins-1, 3, 4, 5, 8, 11, 14, 18, and 19 “tighten” the barrier (sealing TJ proteins), while claudins-2, 10, 15, 16, and 17 have pore-forming properties and thus enhance paracellular permeability (pore-forming TJ proteins for water and ions transport) when upregulated [4,33]. Each of these pore-forming proteins is selective towards the ionic charge type, magnitude, and size, thereby promoting the paracellular transport of ions based on the specific characteristics of claudins [33]. Interestingly, claudin-2, which is abundantly expressed in leaky epithelia, is upregulated in inflammatory situations such as inflammatory bowel disease (IBD), deteriorating inflammation [32]. Hence, a well-orchestrated balance of all claudin isoforms is necessary to preserve functional TJs. Notably, claudin-13 is expressed in rodents but not in humans, while claudins-6, 16, 19, 22, and 24 are not expressed in rodents [26,29]. The roles of claudins-7, 12, and 15 on the intestinal barrier remain elusive, while elimination of claudins-2, 7 and 15 in knockout models results in perturbation of the barrier [3,9]. Most claudins are connected to ZO proteins via their C-terminal PDZ-binding sequence [29].

#### 3.1.2. Occludin

Like claudins, occludin is formed by four transmembrane domains and two extracellular loops, despite the absence of sequence homology between them. The ability of occludin to offer structural integrity to the TJ complex and constitute an integral factor for the functionality of TJ as a barrier is well-established [32]. While the pore pathway permeability seems to be determined majorly by the claudin family, occludin is implicated in the regulation of the leak pathway [8]. Phosphorylation of occludin regulates its function, localization, and interaction with the scaffolding proteins. Specifically, the ser/thr phosphorylated parts are localized in the cellular membrane, while phosphorylation of the intracellular domains occurs less often. The maintenance of TJ integrity presupposes well-regulated interaction between occludin and ZO-1, promoting the stability of the TJ assembly [26]. Suppressed occludin expression is evident in intestinal inflammatory diseases, stressing its pivotal role in preserving barrier integrity [32]. Occludin is mainly involved in cis-homophilic interactions across the cell membrane, and its functions require phosphorylation of the C-terminus and binding to ZO proteins [3,29].

#### 3.1.3. Tricellulin

As noted above, claudins form the barrier of the paracellular route between two adjacent cells. Nonetheless, tricellulin (or marvelD2) is responsible for sealing the transcellular space between three adjacent cells, and it is considered to construct a structurally specialized tricellular TJ assembly. Tricellulin is mainly concentrated at the tricellular contacts, i.e., the points where three epithelial cells converge, forming a central “tube” in tricellular junctions. It comprises four transmembrane domains and two extracellular loops, with the N- and C-termini located in the cytoplasm, similar to the aforementioned transmembrane proteins [1,34]. Notably, it has also been localized at bicellular TJs of the small intestine [34]. This occludin-related protein allows the permeability of large solutes (≤10 kDa), and, interestingly, in cell cultures, tricellulin regulates the passage of macromolecules [1]. In the absence of tricellulin, the organization of both bicellular and tricellular TJs is affected, pointing out its pivotal role in the conservation of organization and stability of paracellular integrity [34]. Like occludin, tricellulin mainly forms cis-homophilic interactions along the cell membrane [3].

#### 3.1.4. Zonula Occludens and Cingulin

The arrangement of the actin cytoskeleton and the interaction between integral transmembrane proteins and cytoskeletal linker proteins is paramount for the dynamic regulation of TJ integrity. Peripheral membrane adaptor/plaque proteins ZO-1, ZO-2, and ZO-3 or TJ protein 1, 2, and 3 and cingulin mediate the connection of the transmembrane proteins to F-actin and myosin of the cytoskeleton and other signaling proteins via strong crosslinks, maintaining TJ shaping and function [26,32,33]. Thus, ZOs and cingulin are considered the scaffold molecules in a TJ [27]. ZOs belong to the membrane-associated guanylate kinase homolog (MAGUKs) superfamily and include various protein binding domains such as three PSD95–DlgA–ZO-1 homology (PDZ) domains, an Src homology-3 (SH3), a leucine-zipper, and an enzymatically inactive GUK domain, in their N-terminal region [9,27,32]. ZOs interact via these domains with various cellular proteins (for scaffold formation, anchoring other TJ proteins to the cytoplasm and cellular signaling) [9,28,32]; Claudins and JAMs bind to two separate PDZ domains, while occludin binds to the GUK domain [28]. The C-terminal regions of ZOs interact with cytoskeletal F-actin, the SH3 domain binds the transcription factor ZONAB, while the formation of dimers between ZOs occurs via the third PDZ domain [27,28]. The exact roles of ZO proteins remain unclear until now. Even though in the absence of ZO-1 in vitro, the paracellular integrity of TJ is preserved, the incorporation of occludin and claudins in the TJ assembly is delayed. By contrast, the deficiency of ZO-2 or ZO-3 has no consequences for the formation of TJ, indicating that ZO-1 has a more crucial role in the control of TJ assembly [26]. ZO-1, as a plaque protein, controls TJ integrity, cell polarization, and reorganization of the cytoskeleton through its multiple interactions with the majority of TJ-related transmembrane and cytoskeletal elements [3,9]. ZO-1 is considered a regulator of the leak pathway [8].

#### 3.1.5. JAMs

The JAM family (JAM 1/A, 2/B, 3/C) constitutes single-span transmembrane proteins with a C-terminal cytoplasmic domain, forming both intra- and extra-cellular interactions essential for the TJ assembly formation and regulation [3,9]. JAM-A has been proved crucial for promoting TJ reassembly and sealing of the paracellular route [9].

### 3.2. Signaling Pathways Involved in the Regulation of TJs

A well-orchestrated regulation of the junctional network and particularly of TJs is of paramount importance due to their role as “gatekeepers” of the paracellular route and their involvement in shaping cellular behavior. The signal transduction processes mediating these effects are bidirectional, meaning that signals are transduced from the TJs to the interior of the cell, modulating gene expression, cell proliferation, and differentiation, and signals are transmitted from the cell interior towards immature and fully developed TJs to direct their dynamics. However, even though TJ-related signaling is still poorly understood due to its complexity, it is clear that these processes are intertwined. Next, the most prominent signaling pathways implicated in the regulation of TJ assembly, abundance, and functionality are discussed, with particular attention to signaling proteins that are considered master regulators of TJs.

#### 3.2.1. AMPK Mediated Regulation of TJs

Among the key players of TJ regulation via TJ assembly/disassembly, the adenosine monophosphate-activated protein kinase (AMPK) has a significant role in promoting the reassembly of impaired TJ complexes. AMPK is a serine/threonine kinase composed of an α catalytic subunit and two regulatory β and γ subunits, which form numerous isoforms in vertebrates [35]. This kinase is a well-established cellular energy sensor of all eukaryotic cells, activated by two principal upstream kinases: Liver Kinase B1 (LKB1) and Ca^2+^/calmodulin-dependent kinase kinase β (CaMKKβ) in response to elevated intracellular AMP (or ADP)/ATP ratios and calcium levels, respectively [36]. The AMPK-regulatory kinase is the transforming growth factor-β-activated kinase 1 (TAK1) [37], which is stimulated via allosteric binding or phosphorylation mediated by various activators [35,36,37]. Besides its key role as a master regulator of energy metabolism [36], the involvement of AMPK in the reinforcement of the paracellular pathway has been proved in various in vivo [38,39,40,41] and in vitro models [42,43,44,45,46,47,48,49]. AMPK is necessary for the early stages of TJ formation, as various signaling pathways are initiated upon its activation, leading to the acceleration of TJ re-assembly. Notably, AMPK activation is not required to preserve TJ integrity but is crucial for protecting and recovering TJ assembly following an induced disruption, as observed in intestinal epithelial Caco-2 monolayers under normal (steady-state) conditions [35].

LKB1, which is stimulated via changes in ATP levels, is a necessary kinase for the maintenance of ZO-1 localization at the plasma membrane [43]. Nevertheless, even though ATP depletion causes TJ delocalization, which is reversed upon ATP repletion [50,51], the AMPK-mediated regulation of TJ assembly is considered independent of ATP levels [36,42]. On the other hand, stimulation of AMPK by CaMKKβ is the most supported mechanism behind the AMPK-mediated regulation of TJs [49]. Nonetheless, the exact ways the activated AMPK favors the TJ assembly have not yet been elucidated, and both Ca^2+^ dependent and independent pathways seem to be involved. As a kinase, AMPK directly phosphorylates various TJs such as ZOs and claudins, and TJ-associated protein-effectors that are implicated in the regulation of TJs, including myosin II regulatory light chain (MLC-2), Protein kinase C (PKC) isoforms of the plasma membrane, cingulin, the polarity scaffold protein Girtin (or G-alpha interacting vesicle-associated, GIV), and the AJ-related scaffolding protein afadin [35,36,37]. Considering that TJs are closely related to AJs via signaling pathways and scaffolding proteins and that stimulation of AMPK is associated with increased TJ and AJ protein expression and stabilization of the apical junction complex, the regulation of TJs by AMPK might be partly related to the AMPK-mediated regulation of AJs [37]. In addition, the implication of the mammalian target of rapamycin (mTOR) signaling [42] and of the transcription factor caudal type homeobox 2 (CDX2) have been identified as essential elements of the AMPK-mediated TJ regulatory pathways [41]. In specific, stimulation of mTOR activity obstructs the AMPK-mediated initiation of TJ assembly, thus AMPK seems to promote TJ functionality, at least in part, via the negative regulation of mTOR [42]. By contrast, AMPK reinforces the intestinal barrier and stimulates epithelial differentiation via the enhancement of CDX2 expression [41].

#### 3.2.2. Myosin Light Chain-2-Mediated Regulation of TJs

The contractility of actomyosin and actin dynamics strictly regulate TJ formation and assembly/disassembly [52]. A principal regulator of TJ permeability properties is the F-actin non-muscle myosin II (NM II) protein of the actomyosin cytoskeleton. NM II is formed of two heavy chains, where interaction with actin occurs, two regulatory light chains and two essential light chains [53]. Intestinal epithelial barrier integrity is directly connected with the levels of the phosphorylated myosin light chain-2 (MLC-2) [53,54,55]. Upon phosphorylation, MLC-2 leads to the unfolding of the actin-binding domain and contraction of the peri junctional actomyosin belt. TJs and AJs, as constituents of the AJC, have direct and indirect interactions with the cytoskeleton [53,56]. As a result, the junctional proteins are internalized, and the barrier integrity is compromised. Under physiological/normal conditions, this mechanism regulates the “opening” of the paracellular route, but over-stimulated contractility of actin filaments due to pathological or pharmacological stimuli compromises the epithelial barrier integrity [55,56]. MLC-2 phosphorylation by the enzymes myosin light chain kinase (MLCK) and Rho-associated coiled coil-containing protein kinase (ROCK), constitutes a TJ-regulating mechanism of crucial importance [53].

MLCK is one of the principal regulators of the leak pathway via the direct phosphorylation of MLC-2, as established by numerous in vivo [57,58,59,60,61] and in vitro studies using intestinal epithelial cell lines [62,63,64,65,66,67,68,69,70,71]. MLCK is a Ca^2+^-calmodulin-dependent serine/threonine kinase involved in the dynamic regulation of actomyosin reorganization and cell contraction in smooth-muscle and non-muscle cells [53,55]. Among the various isoforms encoded by the MYLK genes, MLCK1 (villus) and MLCK2 (crypt-villus axis) are the most abundant kinases in the intestinal epithelium originating from MYLK2 [55,56]. MLCK1 is localized at the peri junctional actomyosin ring of epithelial cells and phosphorylates MLC-2 in response to elevated intracellular Ca^2+^ [68]. MLCK activation is induced by both physiological and pathophysiological stimuli and leads to modest contraction of the perijunctional actomyosin ring, microfilament assembly in the cell periphery, and increase in paracellular permeability via disruption of TJs, but not AJs [52,53,55]. MLCK-mediated MLC-2 phosphorylation in intestinal epithelial monolayers induces re-organization of peri junctional F-actin and significant morphological and biochemical re-distribution of primarily ZO-1 and occludin [56,72].

Rho GTPases compose a family of small signaling G proteins belonging to the Ras superfamily, known to act as molecular switches regulating numerous functions in the IECs [53,55,73]. The Rho signaling pathways are actively involved in the regulation of cell–cell junctions, as extensively reviewed by Terry et al. [73] and Citi et al. [74]. Among the Rho proteins, RhoA (along with Rac 1 and Cdc42) is of utmost significance regarding the control of the actomyosin ring [55,73]. Specifically, in response to extracellular stimuli, RhoA promotes the restructuring of the cytoskeleton via enhancement of F-actin stress fibers formation, their assembly in the center of the cells, and regulation of subcellular focal adhesion [53,55]. RhoA activity is mediated by the binding of GDP (inactivation) and GTP (activation), as dictated by guanine nucleotide exchange factors (GEFs), GTPase-activating proteins (GAPs), and guanine nucleotide dissociation inhibitors (GDIs), when stimulated by extracellular stimuli such as inflammatory cytokines, growth factors, and bacterial products [53,55,73,74]. The fine-tuning of RhoA effectors’ activation is crucial for the normal regulation of TJs [74]. ROCKs are serine/threonine kinases that constitute downstream effectors of Rho GTPases and are composed of a RhoA binding domain, a catalytic domain, and a localization-related domain [53]. ROCK1 is the most abundantly expressed isoform in the intestinal epithelium and is a well-established regulator of the peri junctional actomyosin ring dynamics via direct phosphorylation of MLC-2. In addition, ROCKs mediate the inactivation of the myosin-binding subunit of myosin light chain phosphatase (MLCP), named the myosin phosphatase target subunit 1 (MYPT1). Both events result in markedly elevated phosphorylated MLC-2, leading to the contractility of the cytoskeleton and loss of both TJ and AJ organization [53,55,73,74]. These effects of RhoA/ROCK1-mediated pathways have been well-documented in various in vivo [75,76] and in vitro studies using IECs [77,78,79,80]. Interestingly, in Caco-2 monolayers, both hyperactivation and inactivation of RhoA signaling have been found to increase paracellular permeability, stressing the importance of a balanced pathway activity.

#### 3.2.3. PKC-Mediated Regulation of TJs

Protein kinase C (PKC) constitutes a family of serine/threonine kinases of which the ten isozymes are classified into three groups: the conventional or classical, (α, β1, β2, and γ), novel (δ, ε, η and θ) and atypical (ζ and ι-primates-/λ-mice-) subfamilies [81]. The expression, activation, subcellular distribution, substrates, and mechanism of action vary among the isoforms [82,83]. The stimulation of conventional isoforms (cPKC) is both Ca^2+^ and diacylglycerol (DAG)-dependent, while novel isoforms (nPKC) are DAG- but not Ca^2+^ dependent. Both subfamilies are activated by phosphoserine, while atypical isoforms (aPKC) are both Ca^2+^ and DAG-independent [82,84]. The involvement of PKC signaling in both TJ assembly and disassembly is well-established by various studies, as reviewed by González et al. [82].

Even though the exact underlying mechanisms of TJ regulation by the various PKC isozymes have not yet been fully elucidated [84,85], it is known that PKCs phosphorylate specific TJ proteins, such as occludin and ZO-2 [83]. Several isoenzymes, such as nPKC δ and θ, and aPKC λ and ζ have been associated with TJ proteins at the plasma membrane, but in parallel, PKCs can be activated by other PKC kinases. Thus, TJ regulation can be mediated even by the PKCs not directly located to the TJs [82]. Activation and inhibition of the various isoforms differentially affect TJs, and their transient stimulation is crucial for maintaining a delicate balance [84]. Focusing on the intestinal epithelium, cPKCα on T84 [86] and IEC6 cells, and nPKCθ on Caco-2 cells [87] mediate the opening of TJs [82]. By contrast, cPKCα and nPKCδ on HT-29 and Caco-2 cells promote the TJ assembly upon activation of the pattern recognition receptor Toll-like receptor 2 (TLR-2) [85]. Moreover, nPKCε in T84 cells [88] and aPKCζ in T84 cells promote TJ assembly [82]. In general, nPKCs and aPKCs seem to play a significant role in TJ assembly, while cPKCs in TJ disassembly [82].

The aPKCs-mediated function is less elusive compared to the other two subfamilies. Atypical PKCs are assembled with PAR3 and PAR6 (PDZ domain proteins, localized to TJs), forming an evolutionarily conserved complex essential for cell polarity preservation, along with the Crumbs/Pals/PATJ and Scribble/Disc Large/Lethal Giant Larvae (Lgl) complexes [82]. The binding of PAR3 to JAMs mediates recruitment to TJs, while the binding of PAR6 to the Rho GTPase Cdc42 activates the kinase. Upon the formation of the aPKC-PAR3-PAR6 complexes, late-stage TJ formation and cell polarization are promoted, yet the exact ways of TJ regulation are not known. On the other hand, dephosphorylation of aPKC by protein phosphatase 2A negatively regulates TJs, suggesting that these are opposing signaling pathways [82]. Notably, the initial trigger for junction formation derives from the initiation of cell–cell adhesion by E-cadherin and nectins [84]. The formation of these primary junctions results in the recruitment of AJ and TJ (along with aPKC-PAR3-PAR6) proteins upon the establishment of various protein–protein interactions. This leads to the maturation of the junctional complex into distinct TJs and AJs [82,84].

#### 3.2.4. MAPK-Mediated Regulation of TJs

A great body of evidence supports the implication of another family of kinases, the Mitogen-activated protein kinases (MAPKs), in the regulation of paracellular permeability. MAPKs constitute a family of serine/threonine kinases, members of the CMGC group, which in response to extracellular stimuli control numerous cellular processes, such as proliferation, differentiation, apoptosis, stress responses, and gene transcription [89,90]. MAPKs can be classified into four groups in mammals (known as “conventional MAPKs”): the extracellular signal-regulated kinase 1/2 (ERK1/2) or classical MAP, which upon activation by growth and stress factors, mitogens, cytokines, and other stimuli, regulates cell proliferation, differentiation, and motility. The c-Jun N-terminal kinases 1–3 (JNK 1, JNK 2 and JNK 3) and p38 MAPKs (p38α, p38β, p38γ, and p38δ) are two other groups (together called stress-activated protein kinase pathways), stimulated by the same environmental and cellular stress stimuli, but marginally by growth factors, to mediate mainly cell differentiation and apoptosis. ERK5 (or Big MAPK, BMK) is the fourth group and is activated by both growth factors and stress stimuli to be involved in cell proliferation, differentiation, and cell-cycle progression [82,89,90]. In addition, another group (named “atypical MAPK”) includes the ERK3/ERK4, Nemo-like kinase (NLK), and ERK7, whose biological roles are not clear yet, but it is known that their activation is not MEK-dependent [90].

All MAPKs are involved in specific signaling modules, the so-called MAPK pathways, which occur in two phases: the membranous and the cytoplasmic [82,90]. MAPK pathways are composed of a MKK activator (MKK kinase, MAP3K or MEKK), a MAPK activator (MAPK kinase, MKK, MAP2K or MEK), and a MAPK, which become sequentially activated [90]. Initially, small GTP binding proteins, i.e., Ras, which are close to the growth factor receptors, are stimulated. Then, the MAPK cytoplasmic proteins are successively activated: first, MAP3K is activated and phosphorylates MAP2K, which activates MAPK. In some cases, MAP4K and MAPK-activated protein kinases (MAPKAPK) are also involved [89]. Upon its stimulation, MAPK phosphorylates numerous cytoplasmic and nuclear proteins such as transcription factors and thus can regulate gene expression, including TJ genes [82]. A great many in vitro and in vivo studies have shown that activation of this cascade leads to up- or down-regulation of intestinal epithelial TJ genes, mediated by p38, ERK1/2, and less often by JNK, depending on the stimuli and the experimental model. Hence, upon MAPK stimulation the composition of the TJ complex is altered, leading to either enhanced or impaired barrier integrity via sealing [91,92,93,94,95] or opening of TJs [96,97,98,99,100,101,102,103,104,105], respectively.

#### 3.2.5. Other Signaling Pathways Involved in the Regulation of TJs

Even though the abovementioned pathways are the most prominent in the regulation of the TJs, many other pathways might be involved in the sealing or opening of the paracellular route. Phosphoinositide 3-kinases (PI3K) are enzymes that phosphorylate phosphatidylinositol, converting PI(4,5)P2 to PI(3,4,5)P3. This event results in the translocation of Akt, a ser/thr kinase, from the cytosol to the cell membrane. There, Akt is phosphorylated by the PDK2 and PDK1 kinases, and the activated enzyme stimulates downstream targets such as snail, a transcription factor that suppresses the transcription of E-cadherin, occludin, and claudins via various pathways. Furthermore, PI3K associates with ZO-1 and occludin and can affect the sealing of TJs. PI3K stimulation may induce both an increase and decrease in paracellular permeability. Hence, the exact results of PI3K activation on TJ function seem dependent on the stimuli. Another kinase is the protein kinase A (PKA), an enzyme that, upon activation, leads both to positive and negative regulations of TJs, similar to heterotrimeric G proteins. Interestingly, the evolutionary conserved Crumbs signaling also seems to be involved in the modulation of TJ assembly [82]. Furthermore, the phosphorylation of TJ proteins such as ZO-1, claudin, and occludin on their ser/thr residues positively affects TJ integrity. Dephosphorylation by ser/thr protein phosphatases-2A and -2B inhibits different PKC isoforms, resulting in either assembly or disassembly of TJs [82]. In addition, TJ functionality is also regulated via post-translational modifications such as phosphorylation, glycosylation, and ubiquitination of TJs [106]. The TJ complex is a dynamic structure whose assembly and function continuously alter upon environmental changes and modulation by several stimuli. A cross-talk between these pathways has been revealed, but the exact mechanisms of TJ regulation are yet to be elucidated [84], and a more detailed analysis of the TJ signaling is out of the scope of this study.

### 3.3. Factors Involved in the (Patho)physiological Regulation of TJs

#### 3.3.1. Cytokines and Growth Factor-Mediated Regulation of TJs

As thoroughly discussed, the epithelium plays a vital role as the first line of physical barrier against external antigen-induced malfunction of epithelial TJs, resulting in enhanced paracellular permeability and higher flux across TJs. Increased translocation of commensal microbiota and other luminal antigens through the injured epithelial barrier to the lamina propria stimulates strong inflammatory responses by both epithelial and immune cells [31,107]. Cytokines and chemokines produced by immune cells (innate or adaptive), infiltrating inflammatory cells, or from intestinal epithelial cells themselves, are essential for the regulation of intestinal inflammation and exert physiological and pathological direct effects on TJs [107,108]. Under pathophysiological conditions, various cytokines, principally pro-inflammatory cytokines, induce further dysfunction of the TJ complex, whereas anti-inflammatory cytokines exert protective effects. Tumor necrosis factor α (TNF-α), interferon-γ (INF-γ), interleukins-1β, -4, -6, and -13 (IL-1β, IL-4, IL-6, and IL-13) are cytokines known to mediate the “opening” of the paracellular route, whereas others, such as IL-10, IL-17 and transforming growth factor-β (TGF-β), promote TJ integrity and are considered as barrier-protective cytokines [2,31,107,108]. The available information regarding cytokines-mediated TJ regulation is presented in the next paragraph, though the exact intracellular mechanisms remain unclear.

TNF-α is a proinflammatory cytokine mainly secreted by activated macrophages, monocytes, and T cells, with a crucial role in inducing apoptosis and inflammatory cascades in the intestinal epithelium. TNF-α stimulates NF-κB signaling, which results in the activation of the MLCK gene and increased MLCK protein expression, with subsequent phosphorylation of MLC-2. In addition, TNF-α increases the expression of claudin-2 in a phosphatidyl inositol-3 kinase (PI3K)-dependent manner. Thus, this cytokine not only causes TJ disassembly due to contraction of the actomyosin cytoskeleton and decreased expression of claudin-1, occludin, and ZO-1 but also promotes the expression of a pore-forming TJ protein [2,107,108]. INF-γ is another proinflammatory cytokine majorly produced by natural killer cells and other T cells, mainly involved in inflammatory immune responses. INF-γ stimulates RhoA and increases ROCK expression and pMLC-2, leading to elevated cytoskeletal contractility and internalization of TJ proteins (occludin, claudin-1,-4, JAM-A), resulting in disruption of TJs [2]. Additionally, the INF-γ-mediated decrease in claudin-2 expression has been reported, along with the involvement of PI3K or NF-kΒ pathways in the increase in paracellular permeability [107]. Both TNF-α and INF-γ are markedly elevated under intestinal inflammatory conditions, principally in ulcerative colitis (UC) and Crohn’s disease (CD) patients. Notably, the combination of TNF-α and INF-γ presents synergism, via INF-γ-mediated elevation of TNF Receptor 2 (TNFR2, a TNF-α membrane receptor) levels. Moreover, INF-γ has synergistic effects with lymphotoxin-like inducible protein (LIGHT), another member of the TNF family, via a caveolin-1-mediated internalization of occludin [2,107]. In both cases of synergism, the paracellular route “opens” due to cytoskeletal disorganization, mediated by MLCK activation and accumulated pMLC-2 [2].

IL-1β is a proinflammatory cytokine originated by activated macrophages. It has been shown that the increased level of IL-1β present in the intestinal mucosa is positively correlated with the deterioration of CD and IBD [2,107]. IL-1β stimulates MEKK1, with subsequent activation of NF-κB and increased MLCK expression at transcription and protein levels. These events result in decreased occludin expression and pMLC-2-mediated cytoskeletal re-arrangement and thus dysregulation of TJs [2].

IL-4 is a cytokine secreted mainly by T cells, basophils, and mast cells, which is a well-established mediator of allergic responses, among others, induces the differentiation of naϊve helper T-cells into Th2 cells. IL-4 increases paracellular permeability by increasing the expression and localization of claudin-2. Other mechanisms include stimulation of the PI3K pathway and involvement of STAT6 (the principal transcription factor induced by IL-4), though the exact underlying mechanisms are not fully understood [2,107].

IL-6 exhibits both pro- and anti-inflammatory properties and mediates antigen-specific responses during infection. Similar to TNF-α and INF-γ, strikingly increased levels of IL-6 are evident in IBD patients [2]. Whether IL-6 has barrier-protective effects is a matter of debate; however, IL-6 has been found to increase paracellular permeability in vitro and in vivo [107]. The principal mechanism of IL-6-induced TJ dysregulation involves activation of the MEK/ERK and PI3K/Akt signaling pathways upon binding to its cell membrane receptors and initiation of intracellular signaling via gp130 (a signal transducer subunit). As a result, the expression of claudin-2 is upregulated in a Cdx2-dependent manner, and the paracellular permeability is enhanced [2]. By contrast, in other in vitro and in vivo studies, IL-6 appears to exert barrier-protective effects [108]. Thus, the impact of IL-6 on paracellular permeability may differ among the different experimental models [107].

IL-22 belongs to the IL-10 superfamily, mainly originating from hematopoietic and innate lymphoid cells, such as Th17 cells. IL-22 has multiple regulatory roles, such as cell survival and proliferation, tissue regeneration, and inflammation. Due to its beneficial effects, IL-22 is crucial for preserving a well-regulated intestinal barrier function and is assumed to protect from the pathogenesis of IBD, during which high levels of IL-22 are evident [2]. Nonetheless, activation of the IL-22 receptor leads to stimulation of JAK1 and non-receptor protein tyrosine kinase 2 (Tyk2). The subsequent phosphorylation of STAT-1, -3, and -5 leads to the upregulation of claudin-2 expression and increased paracellular permeability to ions [108,109].

IL-13 is an immunoregulatory cytokine, majorly secreted by Th2, natural killer and mast cells, among others. IL-13 is markedly elevated under inflammatory conditions, such as UC and CD. This cytokine increases claudin-2 expression, probably via a JAK/STAT6/PI3K/Akt-dependent pathway [2,110]. Collectively, even though the cytokines above mediate the increase in paracellular permeability following various pathways, a common mechanism involves the promotion of the pore-forming claudin-2 gene and protein expression [8].

In contrast to the proinflammatory cytokines, specific cytokines can protect the epithelial TJ barrier against disruption and subsequent intestinal inflammation. IL-10 is an anti-inflammatory cytokine predominantly produced by T cells in response to IL-4. IL-10 can combat the effects of TNF-α and INF-γ on the intestinal barrier and is essential for protecting barrier integrity against TJ dysfunction [2,107,108]. Moreover, knockdown of the IL-10 gene disrupts the intestinal barrier under physiological conditions and leads to further impairment under inflammation [108].

IL-17 is a cytokine produced majorly in Th17 cells, highly involved in tissue inflammation and the pathogenesis of autoimmune disorders. Inhibition of IL-17 signaling results in the deterioration of IBD via mislocalization of TJs and increase in paracellular permeability. IL-17 has been found to decrease paracellular permeability due to increased claudin-1 and -2 expression, despite their opposing effects, and combats the TNF-α-induced occludin rearrangement [2,108]. The underlying mechanism of the increased claudin-1 expression is unknown, while the upregulation of claudin-2 is MEK/ERK1-dependent [2,107].

Moreover, even though IL-11 has been reported to exert barrier-protective effects in vivo, the precise explanation of its direct action on intestinal epithelium is not known [107]. In addition, IL-15 seems to enhance TJ integrity, via increased expression and localization of ZO-1, ZO-2, claudin-1, and claudin-2, along with elevated occludin phosphorylation [107]. IL-33 effects are still under discussion, as there are contradictory results regarding the regulation of TJs, depending on the studied model and the examined concentrations [15,108,111]. Nonetheless, reinforcement of TJs in vitro using T84 monolayers seems to occur in an ERK1/2-dependent pathway [15].

Another pleiotropic, anti-inflammatory cytokine that enhances intestinal epithelial TJ functionality is the growth factor TGF-β of the TGF superfamily. TGF-β decreases paracellular permeability under normal conditions and counteracts the effects of INF-γ, highly likely via MEK/ERK- or PKC-dependent signaling. On the other hand, the effects of transforming growth factor-alpha (TGF-α), a member of the epidermal growth factor (EGF) family, on intestinal TJ are controversial [2,107]. Finally, the EGF exerts barrier protection via stimulation of PKC and MEK/ERK signaling pathways [2].

Overall, it is clear that the epithelial barrier integrity can be positively or negatively affected by cytokines and chemokines derived from both immune and intestinal epithelial cells. These alterations are, at least in part, mediated by changes in paracellular permeability due to modifications in TJ expression and localization. Hence, since cytokines have a key role in the maintenance or disruption of the intestinal barrier’s integrity, factors stimulating or halting their release, such as pathogens and anti-inflammatory agents, respectively, can modulate the integrity of the epithelial layer.

#### 3.3.2. Commensal Microbiota and Their Role in TJ Regulation

##### The Gut Flora

Besides the cytokine-mediated regulation of TJ functionality, another key contributor to the regulation of the epithelial barrier is the gut flora. Vertebrates accommodate immense amounts of naturally occurring microorganisms, collectively referred to as microbiota, principally harbored in the gut and considered non-pathogenic [7,13,112]. The importance of the commensal microbiota for health maintenance is now well-recognized [113]. This is no surprise as the gut microbiome contains 150 times the genetic material of the human body [114]. The gut flora communicates with other systems through its metabolome, affecting their function and development [113]. The gut flora in healthy adults is a complex ecosystem composed of approximately 10^13^–10^14^ bacteria, viruses, fungi, archaea, and less often protozoa and helminths [3,7,113,115]. Bacteria constitute the most studied components of the human microbiota, with more than 250 different species present in the digestive tract [3,113]. Among them, Firmicutes (60–80%) and Bacteroidetes (20–40%) are the most abundant, with Actinobacteria, Proteobacteria, Fusobacteria, Spirochaetes, Verrucomicrobia, and Lentisphaerae also present [3,7,112]. Notably, the bacterial communities of the mucosal layer are less populated and diverse than the luminal compartment [7]. The human gut microbiota composition varies according to anatomical location and depends on the host’s genetics [3]. Even though each individual seems to have a specific ecosystem, considering that their microbiome is heritable, 30–40% of commensal bacterial species are common within the general population, indicating the existence of a core microbiome or functional core [7,13,112]. Initially, gut flora is shaped by maternal transmission during birth, enhancing the development of the immune system early in life. However, it is highly affected by environmental factors that impact lifestyle such as diet, stress, socioeconomic state, and geography, but also by the medical and immune status, infections due to pathogens, and other diseases [13,112]. Nonetheless, even though this composition fluctuates over time, it is characterized by relative resilience [3,114].

A particularly mutualistic relationship has been established between commensal microbiota communities and the host, and their co-existence is critical for health [3]. The presence of gut flora is pivotal for numerous intestinal and systemic physiological functions, namely the metabolism of food for energy and nutrient extraction and preservation of optimal metabolic homeostasis [3,7,114]. Gut microbes regulate innate and adaptive immunity thus contributing to the preservation of a robust immune system and have the ability to promote both health and numerous dysbiosis-related mucosal diseases [14,116]. There is a bidirectional interaction between intestinal flora and the host immune system: the gut microbiome promotes the immune system, directly and indirectly, providing the immune system with appropriate signals to ensure tolerance towards the microorganisms [13]. Direct immunomodulation is exerted by bacterial cell surface structures and secreted metabolites and other effectors such as extracellular vesicles of the microbes [117,118,119]. In specific, commensal bacteria contain surface components that are microbial-associated molecular patterns (MAMPs), e.g., flagella, surface layer proteins (SLPs), pili, and LPS. Upon their recognition by epithelial innate immune sensors named pattern recognition receptors (PRRs), which are also expressed by dendritic cells (DCs), MAMPs stimulate signaling pathways on the IECs, including NF-κΒ, MAPK, and PPARγ-dependent mechanisms. Moreover, MAMPs elicit cytokine and chemokine release upon the regulation of a cellular protease-dependent signaling cascade, resulting in the suppression of inflammation and protection of epithelial integrity [17,120]. In addition, gut microbiota-mediated indirect immunomodulation occurs via food metabolism. Gut flora metabolites derived from nutrient metabolisms, such as short-chain fatty acids (SCFAs), possess immunoregulatory properties, e.g., via stimulation of free fatty acid receptors on immune cells [16,121]. Concurrently, the immune system responds to and modifies the affluence and colonization of the commensal microbiota and pathogens [13,112]. Even though the effect of adaptive immune response on gut flora is obscure, it is well-established that DCs sample intestinal contents, including MAMPs, by extending dendrites expressing TLRs to the lumen and thus determine the type of adaptive immune response to microbes [13,120]. However, mutualistic relations between adaptive immune cells and commensals have been described [13]. Notably, several host epithelium-associated factors have been demonstrated as gut flora modulators. Briefly, microbiota shaping occurs majorly via the PRRs, AMPs, the mucus barrier, sIgAs, the epithelial microvilli, epithelial metabolism, the oxygen barrier, and host microRNAs. With these shaping factors, the intestinal epithelial cells select the residing microbes and influence the composition of the microbial populations [16]. Among the most crucial microbiota-shaping host factors, are the PRRs. The enterocytes express various membrane-bound or cytosolic PRRs, including TLRs, C-type lectin-like receptors (CLRs), nucleotide-binding oligomerization domain (NOD)-like receptors (NLRs), retinoic acid-inducible gene-I (RIG-I)-like receptors (RLRs), and absent-in-melanoma (AIM)-like receptors (ALRs) [13,16,120]. Bacterial MAMPs are sensed by the PRRs and stimulate immune responses including the production of AMPs, sIgA transportation, and immunocyte recruitment. Moreover, the transmembrane and gel-forming mucins comprising the mucus are involved in host-microbes communication by forming a protective barrier, acting as luminal sensors for gut immunity, and providing a habitat for residing microbes. In addition, sIgAs produced by plasma cells promote both pathogen clearance and commensal adherence to the mucus and the epithelial layer, while the epithelial microvilli act as an electrostatic barrier that obstructs microbe adhesion. Furthermore, the competition between host and microbes for nutrients and the levels of luminal oxygen also shape gut microbiota. Finally, epithelial cell-derived microRNAs affect gut microbes’ growth and gene expression, thus contributing to microbiota shaping [16].

##### Dysbiosis

Dysbiosis/dysbacteriosis refers to the unbalanced composition, abundance, diversity, and activity of the microbiota populations building the gut flora [3,7]. Dysbiosis entails an elevated abundance of the LPS-containing bacteria rather than the less LPS-potent Bacteroides and is involved in the pathogenesis and progression of intestinal and systemic disorders [7,112,116]. The dysregulated gut flora is less abundant with health-promoting bacteria, e.g., SCFA-producing bacteria such as *F. prausnitzii*, *Bifidobacterium* spp., *A. muciniphila*, and *Bacteroides* spp. [7]. During dysbiosis, the accumulation of endotoxins and the lack of beneficial microbiota lead to impaired intestinal barrier integrity and the so-called ‘leaky gut,’ subsequently triggering inflammatory responses [7]. Consecutively, the translocation of even more pro-inflammatory LPS into the lamina propria and finally to the circulation aggravates the pathological conditions [3]. Imbalances in the intestinal communities result in inflammatory diseases of the intestine as well as of organs at distal sites [122]. Dysbiosis-associated intestinal disorders include IBD (i.e., UC and CD), celiac disease, IBS, colorectal cancer (CRC), and necrotizing enterocolitis [112,116]. Extra-intestinal disorders include metabolic syndrome, obesity, type 2 diabetes, non-alcoholic fatty liver disease (NAFLD) including steatohepatitis (NASH), alcoholic liver disease including alcoholic steatohepatitis (ASH), cardiovascular disease, and allergy [7,112,114]. In addition, microbial dysbiosis is observed in central nervous system disorders via the gut–brain axis, namely autism spectrum disorder, Alzheimer’s disease, cognitive dysfunction, Parkinson’s disease, chronic depression, and anxiety (stress-related disorders) [112]. These diseases are triggered by pathogenic bacteria, as well as by otherwise harmless commensals and pathogenic commensals, whose population is controlled by the host’s gut ecosystem and immune and metabolic states. Thereby, disturbance of its homeostasis, e.g., due to the use of broad-spectrum antibiotics, can result in life-threatening dysbiosis [122].

##### Regulation of TJ via the Gut Flora

Contrary to intestinal inflammation, which is a pivotal contributor to increased paracellular permeability, gut commensals constitute a principal factor positively affecting intestinal epithelial integrity [7,115]. Indeed, numerous in vitro and in vivo studies support the notion that probiotic commensals such as *Bacteroides fragilis*, *Lactobacillus reuteri*, *F. prausnitzii*, *Bacteroidales* spp., and *Bifidobacterium* spp., reinforce the intestinal epithelial barrier under physiological and pathophysiological conditions [14,123,124,125,126,127,128,129,130]. These barrier-protective effects result from alterations in the TJ gene and protein expression and localization, as induced by the stimulation or inhibition of proteins involved in TJ regulation. Among these cell-signaling proteins are the PKC, ROCK, MLCK, GTPases, and ERK1/2. [9]. In addition, gut microbes can suppress the noxious effects of pro-inflammatory cytokines known for disrupting TJs, e.g., TNFα and IFNγ, and promote cytokines involved in TJ reinforcement such as IL-6 [7,9]. These effects are exerted directly via cell structural components such as pili and indirectly by bacterial metabolites, including SCFAs, indole, and extracellular proteins and are strain-specific [17,131].

Bacterial surface component-mediated regulation of TJs.

As mentioned above, commensal bacteria contain surface components that are MAMPs and upon their recognition by PRRs, MAMPs regulate signaling pathways involved in inflammatory and apoptotic responses, reinforcing the gut barrier’s functionality [17]. Incubation of Caco-2 monolayers with *A. muciniphila* or Amuc_1100, a pili-like protein located in the bacterial outer cell membrane, promotes TJ formation as evidenced by the increased transepithelial electrical resistance (TEER) values [117]. Consistently, treatment of high-fat diet (HFD)-fed mice with Amuc_1100 reinforces the mechanical barrier via increasing claudin-3 and occludin gene expression in a TLR2-dependent manner [132].

Bacterial effectors and metabolite-mediated regulation of TJs.

The regulatory effects on TJs by probiotic commensals seem to be majorly exerted by their metabolites. An extracellular protein secreted by *B. infantis* reinforces the TJ complex in T84 monolayers via downregulation of claudin-2 and upregulation of ZO-1 and occludin, upon up- and down-regulation of pERK and pp38, i.e., via stimulation of MAPK signaling. In addition, treatment of the monolayers with the same *B. infantis* conditioned medium (BiCM) attenuates TEER decrease, and TJ re-arrangements as induced by TNF-α and INF-γ. In the same study, supplementation of IL-10-deficient mice with BiCM alleviates inflammation and normalizes mannitol flux [133]. Furthermore, indole, a metabolite of several commensal tryptophanase-possessing bacteria (e.g., *Bacteroides ovatus*, *Clostridium bifermentans*), is a quorum-sensing molecule used as intestinal symbiotic bacteria signal [134]. Exposure of HCT-8 cells to indole strengthens epithelial barrier functionality via the downregulation of claudin-2 and upregulation of ZO-1, -3, and -4 protein levels with a corresponding TEER increase. These effects are partly mediated by the stimulation of pathways involved in the formation of TJs and cytoskeleton arrangement such as PI3K/Akt and PKC pathways [119]. In addition, the administration of indole in germ-free mice which appeared with decreased cell–cell junctions gene expression, resulted in the elevation of ZO-1, claudin-7, and occludin mRNA levels [134]. Moreover, indole 3-propionic acid, an indole metabolite and ligand of the pregnane X receptor involved in the regulation of intestinal permeability via TLR4 has also been shown to promote TJ gene expression and to decrease TNF-α in mice [118].

Other structures secreted by the gut microbiota are the extracellular vesicles (EVs) or outer membrane vesicles (OMVs), which are produced by Gram-negative bacteria, and are lipid bilayer vesicles associated with bacteria-host communication [135,136]. LPS-containing OMVs originating from dysbiotic bacteria induce a strong inflammatory response and promote epithelial TJs disruption via various pathways, including TAK-1 and MLCK activation by TLR4/MyD88- and NF-κΒ p65/p50-signaling transduction [57], and hyperactivation of Ca^2+^-activated Cl^-^ channels and epithelial Na^+^ channels [137]. However, the OMVs of beneficial commensal bacteria are considered positive regulators of the TJ complex. Supplementation of HFD-fed mice with *A. muciniphila*-originated EVs (AmEVs) enhances ZO-1, occludin, and claudin-5 abundance and reduces FITC-D permeability. In the same study, treatment of LPS-challenged Caco-2 monolayers with the AmEVs saves TJ integrity as indicated by the increased TEER values, decreased fluorescein isothiocyanate–dextran (FITC-D) flux, and upregulated occludin protein levels via stimulation of AMPK [138]. In addition, the OMVs and soluble factors of *Escherichia coli* ECOR63 and *E. coli* Nissle 1917 (EcN) have been reported to reduce claudin-2 and elevate ZO-1 and claudin-14 mRNA and protein levels with parallel TEER increases in Caco-2 and T84 monolayers [139]. Furthermore, TcpC, a bacterial factor secreted by EcN, increases the TEER of HT-29/B6 cells and upregulates claudin-14 via PKCζ and ERK1/2 pathways [140]. Furthermore, treatment of DSS-challenged mice with *F. prausnitzii* supernatant decreased the paracellular permeability of ^51^Cr-EDTA, thus enhancing intestinal barrier function [141]. Finally, pre-treatment of INF-γ-challenged enteroids with LGG-conditioned media prevents disturbances in ZO-1 and occludin gene expression and abundance and decreased FITC-D flux. Notably, this barrier-protective effect is attributed to LGG-secreted proteins and is independent of its action against apoptosis. Similarly, pre-incubation of colonoids exposed to fecal supernatants from IBD patients with the LGG supernatant exhibits FITC-D retention similar to control levels [142].

Health-associated commensal bacteria metabolize non-digestible dietary carbohydrates, i.e., dietary fibers, resistant starch, and less often dietary and endogenous proteins [143]. The fermentation end-products produced in the colonic lumen are SCFAs which are organic acids, principally butyrate, acetate, and propionate, and constitute the preferential energy source of colonocytes [7]. Butyrate offers 60–70% of the energy requirements and is a well-recognized positive regulator of the TJ complex, an immune system modulator, and an inflammation suppressor [17,143]. Nonetheless, it is not the most abundant SCFAs produced, as it corresponds to almost 15% of total SCFAs, while acetate and propionate correspond to approximately 60% and 25%, respectively [143]. Butyrate-producing bacteria include mainly the phylum Firmicutes (e.g., genus *Roseburia*, *F. prausnitzii*, *Eubacterium hallii, E. rectalis*). In addition, other bacteria such as *Bifidobacterium* and *A. muciniphila* produce as final fermentation products propionate and acetate, which are further used by bacteria capable of butyrate production. For instance, these so-called cross-feeding interactions have been identified between Bifidobacteria, Lactobacilli, and butyrate-producing colon bacteria [7]. Butyrate is involved in the direct decrease in paracellular permeability via the promotion of TJ expression (on both a transcriptional and protein level), TJ proteins localization, and TJ assembly/disassembly both in vitro [65,144,145,146,147,148,149,150,151,152] and in vivo [153,154,155]. One of the most prominent mechanisms of TJ reinforcement is the AMPK-mediated acceleration of TJ assembly. Incubation of Caco-2 monolayers with butyrate during a calcium-switch assay leads to activation of the CaMKKβ pathway and thus increase in pAMPK, resulting in inhibition of MLC-2 phosphorylation and stimulation of PKCβ2, ultimately promoting the re-assembly of TJs [65]. This is in accordance with previous studies on the Caco-2 cell line demonstrating that butyrate promotes epithelial integrity via the AMPK-dependent TJ re-assembly, a necessary mechanism for the attenuation of increased paracellular permeability induced by ethanol [147,156]. Another study on LPS-challenged IPEC-J2 monolayers revealed that butyrate pretreatment rescues epithelial integrity by upregulation of claudin-3 and -4 expression via mechanisms involving the Akt/mTOR-mediated protein synthesis and AMPK-related regulation of cellular energy status [157]. Accordingly, incubation of Caco-2 cells with sodium butyrate enhances claudin-3 expression in an Akt-dependent pathway upon stimulation of the G protein-coupled receptor 109A [154]. Besides, butyrate enhances barrier formation, as indicated by the increased TEER values of T84 cells and decreased pore-forming claudin-2 mRNA on both T84 and Caco-2 cells. The repression of claudin-2 is mediated by the promotion of the epithelial anti-inflammatory IL-10 receptor α subunit (IL-10RA). IL-10RA is a significant factor for barrier formation and its expression and function require stimulation of STAT3 and inhibition of histone deacetylase (HDAC) [149]. In addition, butyrate has been shown to elevate lipoxygenase mRNA levels through histone hyperacetylation and consecutive regulation of gene transcription, resulting in a TEER increase in Caco-2 monolayers [145]. These results agree with a previous study identifying HDAC inhibitors, such as butyrate, as positive regulators of the TJ complex via the upregulation of TJ gene expression in various cell lines [144]. Notably, acetate and propionate can also reinforce TJ function in vivo and in vitro, albeit to a lesser extent than butyrate [152,156,158]. Nevertheless, the accumulation of high SCFA levels, e.g., due to the rapid fermentation of dietary fibers, is toxic for the colonocytes due to the induction of apoptosis and impairment of the intestinal barrier [146]. Finally, Urolithin A (UroA) is another metabolite generated by commensal microbiota and originates from the ellagic acid (EA) found in berries and pomegranates. UroA and its analogue UAS03 upregulate claudin-4 protein levels via an aryl hydrocarbon receptor (AhR)-nuclear factor erythroid 2-related factor 2 (Nrf2)-dependent signaling in HT-29 monolayers. In the same study, supplementation of 2,4,6-trinitrobenzene sulfonic acid-induced colitis mice with UroA/UAS03 decreases FITC-D permeability via stimulation of the AhR-Nrf2 signaling [159].

A well-orchestrated dialogue between the host and gut microbiota is pivotal for overall health, considering their strong interplay. The host responds to and modulates microbial populations residing in the intestine, preserving the balance between health-beneficial and pathogenic bacteria. In parallel, gut flora microbes influence intestinal and systemic immunity and facilitate the maintenance of a homeostatic organism. Among the critical roles of the microbiota is the reinforcement of the epithelial barrier. Probiotic gut commensals positively modulate the formation and function of TJs via their cell-surface structures and metabolites and thus preserve the intestinal barrier’s integrity. The enhanced barrier functionality occurs under both homeostatic and leaky-gut conditions and is evident both in in vitro and in vivo models. Hence, factors known to shape intestinal microbiota populations and reverse dysbiosis, i.e., prebiotics, can significantly contribute to intestinal barrier reinforcement and to the overall host health.

## 4. Non-Digestible Oligosaccharides: Their Role in TJ Modulation

Manipulation of commensal microbiota via prebiotic supplementation has emerged as an indirect strategy of intestinal barrier reinforcement. NDOs have gained tremendous attention in recent years due to their multiple health-beneficial properties [19,20]. NDOs are chemically stable carbohydrate polymer compounds and are well-established dietary fibers and prebiotics [160,161]. Usually, NDOs consist of 3 to 10 sugar moieties, though the degree of polymerization (DP) ranges from 2 to 60 for some NDOs, e.g., lactulose and chicory inulin, respectively [21]. The term prebiotics refers to dietary substances that selectively stimulate the growth and/or activity of one or a limited number of bacteria already resident in the colon and induce an overall favorable modulation of the gut microflora, restoring dysregulated microbial communities and thus promoting gut homeostasis and host health [162,163,164]. Once ingested, NDOs resist hydrolysis by salivary and digestive enzymes of the GI tract and reach the caecum and colon intact, where they are fermented by the gut microbiota, resulting in mainly SCFAs, particularly butyrate, acetate, and propionate [19]. As discussed previously, both the SCFAs and commensal bacteria can influence the function of the TJ complex through immunomodulatory and anti-inflammatory activities or/and via direct stimulation of signaling pathways involved in the regulation of TJs. Since the NDOs beneficially alter the commensal microbiota populations and lead to increased SCFA levels, they can protect and reinforce the epithelial integrity in a microbiota-dependent manner.

**Figure 2:**
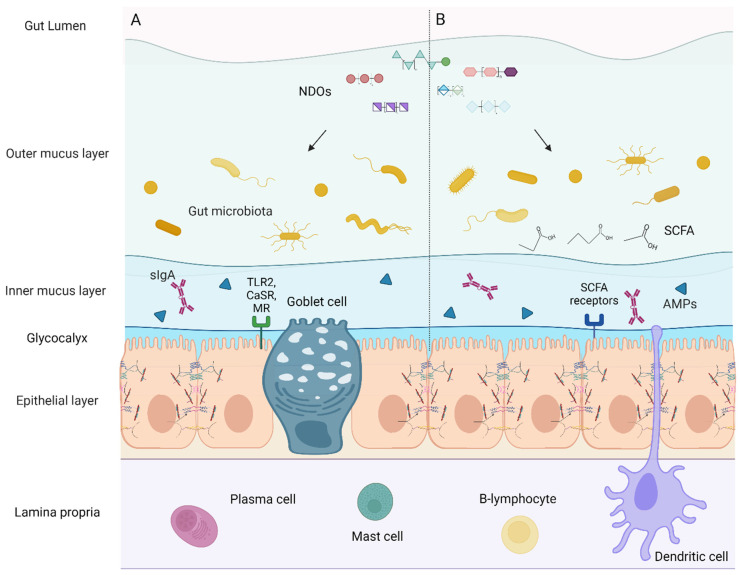
NDO-mediated regulation of TJs: The key elements of the mucosal barrier from the luminal to the basolateral surface are (1) the gut microbiota, (2) the outer and inner mucus layers containing plasma cell-originated sIgA, and the unstirred water layer, i.e., glycocalyx, (3) the epithelial monolayer mainly built of absorptive enterocytes, enteroendocrine cells, mucin-producing goblet cells, and AMP-producing Paneth cells, (4) the lamina propria comprised of IgA and cytokine-secreting innate and adaptive immune cells. Intercellular TJs connecting adjacent epithelial cells strictly regulate paracellular permeability and gut barrier integrity. The NDOs, once ingested, reach the colon intact and exert microbiota-independent (**A**) and -dependent (**B**) effects on the epithelial TJ complexes, thus modulating epithelial integrity. The microbiota-independent regulation of TJs results from direct interactions between NDOs and cell surface receptors of IECs or innate immune cells present in the lamina propria. Thereby, the NDOs reinforce the epithelial barrier via direct induction of host kinome responses (i.e., of the complete set of protein kinases encoded in an organism’s genome) and protect the integrity of the monolayer against noxious stimuli known for impairing TJ abundance and localization upon suppression of inflammatory responses (**A**). Concurrently, the NDOs enhance the mucosal barrier due to their prebiotic capability: First, they stimulate the growth of health-beneficial bacteria, which positively regulate TJs directly via their cell surface structures and excreted effectors. Second, NDO supplementation results in elevated concentrations of SCFAs (principally butyrate, acetate, propionate), which are the primary metabolites produced upon NDO fermentation by the gut commensals and are potent regulators of the epithelial TJs. Third, NDOs restore dysbiosis, a condition accompanied by leaky gut syndrome due to impaired TJ functionality. Re-establishment of balanced gut flora populations due to the decrease in pathogenic bacteria and the increase in beneficial ones leads to alleviation of intestinal inflammation and of the subsequent disruption of the epithelial barrier. Consecutively, NDOs enhance and protect mucosal barrier function under normal and pathological conditions, respectively (**B**). Overall, NDOs can positively affect TJ localization and expression via their direct interactions with cell surface receptors and through their fermentation products. Created with BioRender.com. AJ, Adherens junctions; AMP, antimicrobial peptide; IECs, Intestinal epithelial cells; NDO, Non-digestible oligosaccharide; SCFA, short-chain fatty acids; sIgA, Secretory immunoglobulin A; TJ, Tight junctions.

Interestingly, several NDOs have been shown to directly interact with IECs and immune cells as intact structures, i.e., prior to their metabolism by the gut microbes [19,161]. As a result, these oligosaccharides directly influence TJ abundance and dynamics via stimulation of pathways involved in TJ regulation and immunomodulation, thus in a microbiota-independent manner. Overall, NDOs impact paracellular permeability via two major pathways: (1) microbiota-dependently, via their fermentation products and their ability to combat dysbiosis, and (2) microbiota-independently, as intact compounds that possibly interact with cell surface receptors (Figure 2). This review concentrates mainly on NDOs with DP < 10 and their effects on intestinal epithelial TJs. Special attention is given to the mechanisms underlying the gut flora-independent effects, hoping to shed some light on the complex signaling pathways by which these oligosaccharides impact the TJs.

### 4.1. Fructooligosaccharides

#### 4.1.1. Structure and Sources

**Figure 3:**
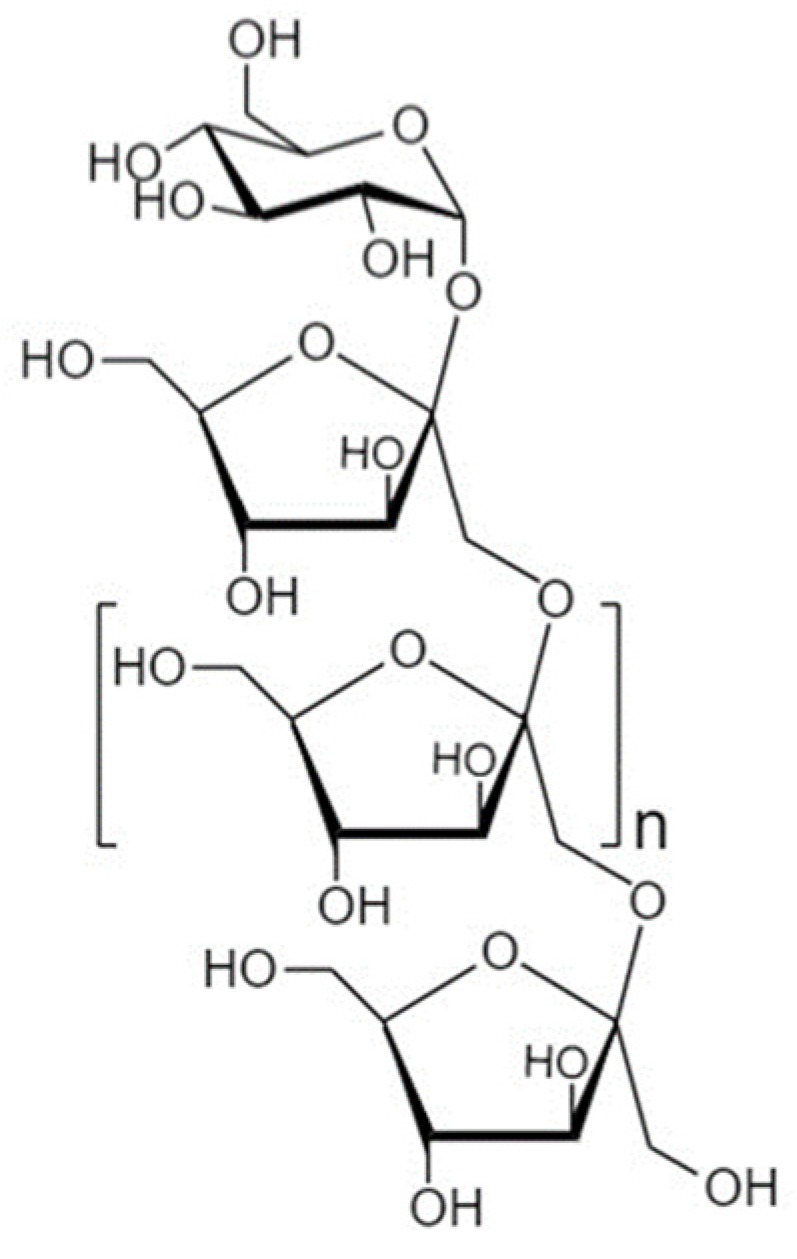
FOS structure: the main components of FOS are n 2,1- or 2,6-linked β-d-fructose monomers and a terminal 1,2-linked α-d-glucose. FOS, fructooligosaccharides.

Fructooligosaccharides (FOS), also named oligo-fructans or oligofructose, are common components of a healthy diet and have drawn significant attention due to their prebiotic functionalities [165]. FOS belong to the family of fructans, which are carbohydrates composed of D-fructose monomers sometimes linked via β-(2→6) glycosidic bonds (Levan type), but usually with β-(2→1) linkages and a terminal α-(1→2) linked D-glucose residue [81,161,165,166] (Figure 3). FOS have a degree of polymerization (DP) 3–9, while fructans with a DP > 10 are termed inulin or long-chain FOS [23,166,167]. FOS are abundantly synthesized in higher plants where sucrose, a glucose-fructose disaccharide, is transformed into fructose by a transfructosylating enzyme [161,168]. The most common natural sources include chicory root, artichoke, onions, garlic, asparagus, leeks, and wheat [167,169]. These compounds can be obtained either by extraction from plants or by enzymatic manufacture, enzymatic or acidic degradation of inulin, and action of fructosyltransferases on fructose [161,170,171]. FOS are resistant to hydrolysis by human alimentary digestive enzymes but are sensitive to hydrolysis by colonic bacteria [81,170,172]. Nonetheless, depending on the DP and their physicochemical properties, differences in fermentability (lower DP results in faster fermentation rates) and transit time appear [169,173].

#### 4.1.2. Microbiota-Independent Effects on TJ

Numerous in vitro studies have established the FOS-mediated barrier protective effects (Table 1). Despite the elucidation of specific underlying mechanisms by which FOS operate on TJs, the lack of in-depth mechanistic studies has halted their clear categorization.

##### *FOS* *Directly Modulate TJs via Stimulation of Intracellular Calcium Signaling*

The calcium-sensing receptor (CaSR) is a G protein-coupled (Gq) receptor expressed in IECs. Upon extracellular Ca^2+^-mediated stimulation of CaSR, various responses occur, such as the upregulation of D-myo-inositol 1,4,5-trisphosphate (IP3) production by phospholipase C (PLC). IP3 takes part in intracellular signal transduction pathways where it serves as a second messenger, mediating the release of intracellular Ca^2+^ ([Ca^2+^]_i_) upon binding to its receptor [174]. A recent study on colonic adenocarcinoma-derived T84 monolayers revealed that FOS directly stimulates CaSR leading to activation of the PLC-IP3 receptor and the release of Ca^2+^ from intracellular sources with a subsequent increase in [Ca^2+^]_i_ levels. In response to [Ca^2+^]i accumulation, the CaMKKP/AMPK pathway is stimulated, resulting in the acceleration of TJ re-assembly. Based on this mechanism, treatment with FOS induces TJ re-localization during a calcium-switch assay, under both LPS-challenged and unchallenged conditions, without altering their expression. Interestingly, the most prominent effect is exerted with a basolateral application, indicating that the interaction between FOS and the basolaterally located CaSR is of high significance [166]. Moreover, higher FOS concentrations fail to induce AMPK phosphorylation, pointing out that CaSR/AMPK desensitization phenomena may occur, along with the fact that FOS interact with distinct receptor types depending on the examined concentration [166]. By contrast, Suzuki et al. reported that apically added FOS increases the paracellular permeability of Caco-2 cells, and this effect is highly correlated with paracellular Ca^2+^ transport and concentration-dependent elevation of [Ca^2+^]i levels. The mechanism proposed involves direct stimulation of the IECs by FOS, which results in increased [Ca^2+^]i and subsequent activation of MLCK [175]. As previously discussed, stimulation of the MLCK/MLC-2 pathway leads to condensation of the actin microfilaments and physiological opening of the TJ pore, explaining the increased intracellular Ca^2+^ absorption through the physiological control of TJs [56,175]. Notably, these effects are not evident with the basolateral application, implying the presence of an apically located sensory system, especially considering that NDOs such as FOS cannot permeate through the cell membrane [175]. However, the FOS-mediated increase in paracellular permeability in vitro may result from the crosstalk between various signaling pathways. For instance, knowing that FOS activates AMPK, which has been found to enhance the integrity of the intestinal barrier via the inactivation of MLCK/MLC-2 and PKCβ2 phosphorylation [65], an interplay between AMPK-MLCK-PKC might be involved in the FOS-induced opening or sealing of TJs.

##### *FOS* *Protect TJ Integrity via TLR2/PKC/MAPK Associated Pathways*

FOS, as β-(2→1) fructans, are established ligands of the pattern recognition receptor TLR2, which is implicated in the regulation of the innate immune system. Based on a study using human peripheral blood mononuclear cells, shorter-chain β-(2→1) fructans (DP 2–10) favor a more anti-inflammatory balance (increased IL-10/IL-12 ratio) compared to long-chain fructans (DP up to 60) [176]. Activation of TLR2 plays a crucial role in physiological TJ regulation and exerts barrier-protective effects through activation of PKCα/δ in both T84 and Caco-2 monolayers [85]. These atypical PKC isoforms interact with the PI3K/Akt pathway upon recruitment of the cytoplasmic adaptor protein MyD88 and seal the TJ in a ZO-1 mediated manner, as indicated by in vivo studies [177,178]. Based on an in vitro study using T84 cells, FOS pre-treatment prevents the phorbol 12-myristate 13-acetate (PMA)-mediated disruption of TJ integrity upon stimulation of TLR2 and interference with PKC signaling. Nonetheless, the exact PKC isoforms involved in the observed effects were not investigated. In the same study, FOS post-treatment upon the PMA challenge does not exert any recovery effects, indicating that the timing of treatment determines the ultimately observed effects [179]. Moreover, Wu et al. demonstrated that FOS pre-treatment of Caco-2Bbe1 cells under enterohemorrhagic *E*. *coli* (EHEC) challenge protects TJ abundance and localization [81]. Investigation of the kinome response revealed that FOS pre-treatment induces the phosphorylation of several kinases involved in MAPK and TLR pathways known to mediate TJ regulation. FOS seem to activate TLR signaling leading to the activation of PKCδ, which in turn induces downstream MAPK pathways. Similarly, FOS protects the barrier function of intestinal organoids via TLR/PKCδ pathways, yet these effects are not accompanied by stimulation of MAPK signaling [81]. This observation indicates that FOS directly modulates and protects the intestinal barrier via various pathways, depending on the tested model. Nonetheless, PKCδ activity is necessary for the observed effects, since its inhibition halts the barrier protective effects conferred by FOS [81]. Contrary to the above, based on a previous study on EHEC-challenged Caco-2Bbe monolayers, FOS fails to exert any protective effects on TJs and F-actin arrangement [180]. In this case, a possible explanation could be the contribution of the structural differences between FOS formulations used in the different studies.

##### *Other* *Direct Effects of FOS on Epithelial Barrier Integrity*

Recently, Lafontaine et al. demonstrated that treatment of Caco-2 cells with FOS enhances TJ integrity, as evidenced by the increased TEER values. Surprisingly, this effect is not accompanied by altered TJ gene or protein expression [171]. However, according to the same study, FOS upregulates genes involved in ovarian steroidogenesis, arachidonic acid metabolism, and general metabolic pathways. The suggested underlying mechanism explaining the observed barrier reinforcement involves the inducement of compositional alterations of sterols and phospholipids, followed by changes in the cell membrane rigidity-fluidity and stabilization-disruption, respectively [171]. Based on another study in Caco-2 cells, FOS mitigate the deleterious effects of the mycotoxin deoxynivalenol (DON) and rescue the TJ integrity in a concentration-dependent manner, though the underlying mechanisms were not further investigated [23]. Furthermore, numerous studies on IECs demonstrate that FOS directly influence the host kinome activities and suppress TLR-, NF-κB-, and MAPK- inflammatory cascades, mitigate the detrimental effects of TNF-α, INF-γ, LPS, increase the expression of TGF-β and anti-inflammatory cytokines (e.g., IL-10, IL-6), as well as decrease pro-inflammatory cytokine expression or secretion (e.g., IL-12, IL-8) [166,180,181,182]. As mentioned above, FOS directly regulates the signaling of immune cells mainly via TLR2 among the TLRs, favorably altering the IL-10/IL-12 ratio and IL-6 levels in a concentration-dependent manner [176], and exerting anti-inflammatory effects via inducement of PPARγ [182], a nuclear receptor that inhibits the production of inflammatory cytokines by interfering with a TLR4-dependent signaling pathway [183]. Collectively, these results indisputably indicate that intact FOS structures protect the intestinal epithelial barrier via direct interactions with the IECs and modulate innate immune signaling and TJ function. The discrepancies between the observed effects can be explained by differences in FOS formulations, concentrations, time points, and the model used in the different studies.

#### 4.1.3. Microbiota-Dependent Effects on TJ

FOS resist enzymatic hydrolysis and pass through the GI tract until reaching the colon intact, where they are fermented by commensal microbiota [171,172]. Studies on mice models of IBS [163], NASH [184], mucositis [185,186], chronic stress [187], HFD [188], food allergy [189], and enteropathogenic *E. coli* (ETEC)-challenged [190], as well as healthy weaned piglets [170,191], have revealed the beneficial effects of FOS supplementation with regard to barrier integrity. Based on these studies, FOS supplementation restores dysbiotic populations with parallel alleviation of inflammation, leading to attenuation or repair of TJ dysfunction or injury (Table 1). Accordingly, studies implementing the Simulator of the Human Intestinal Microbial Ecosystem (SHIME^®^) demonstrated that fermentation of FOS by the commensal microflora significantly elevates butyrate, acetate, and propionate levels, along with a positive alteration of the prebiotic index (PI), i.e., increased bifidobacteria and/or lactobacilli populations and decreased bacteroides and clostridia [168]. Based on such a setting, FOS manage to decrease paracellular permeability via elevation of SCFAs abundance [192], in agreement with other in vitro studies using FOS fermentation supernatants [193,194].

Even though prophylactic and therapeutic supplementation with FOS indisputably promotes TJ integrity and reinforces the intestinal epithelial barrier, some reports show FOS-mediated increase in paracellular permeability in vivo (Table 1). FOS supplementation of *Salmonella enterica*-infected rats further increases paracellular permeability with corresponding enhanced bacterial translocation and intestinal injury [195]. Based on a later study from the same group, FOS treatment increases the paracellular permeability of healthy rats without alterations in the expression of TJ genes. In contrast, numerous mitochondria-related genes are significantly stimulated, indicating that FOS influences intestinal mucosal energy metabolism [196]. Nonetheless, the observed effects can be attributed to rapid FOS fermentation by the gut microbiota and the fact that rats have higher intestinal permeability than humans. Rapid fermentation of FOS causes excessive production and accumulation of SCFAs in a dose-dependent manner, leading to marked acidification of luminal contents (i.e., drop in pH) and subsequent ATP depletion of colonic epithelial cells, which eventually results in increased paracellular permeability [50,195,197,198].

**Table 1:** Microbiota-dependent and independent effects of FOS on paracellular permeability and/or TJs.

Treatment Characteristics	[FOS]	Model/Experimental Setup	Type of Study	Observed Effects on PP and/or TJs	Type of Effect	References
FOS (Sigma-Aldrich, St. Louis, MO, USA)	0.1 mg/mL	T84 monolayers	In vitro	Ca^2+^ switch assay under normal and LPS-challenged conditions: ↑ TEER/acceleration of TJ re-assembly (better effect with basolateral application)	MID	[166]
				Re-localization of ZO-1, occludin and claudin-1, no alterations on TJ proteins expression		
FOS (Nutraflora^®^, Nutrition GTC, Golden, CO, USA ), DP 2–9	10% *w*/*v*	EHEC-exposed Caco-2Bbe1 monolayers	In vitro	Pre-incubation/ challenged cells: ↑ TEER, redistribution of ZO-1, ↑ mRNA ZO-1, ↑ occludin protein but not mRNA	MID	[81]
				Unchallenged cells: no effect on TEER, ↑ mRNA & protein ZO-1, ↑ occludin protein but not mRNA, no effect on claudin-1		
FOS (Nutraflora^®^), DP 2–9	10% *w*/*v*	Duodenal organoids	In vitro	Pre-incubation/challenged organoids: ↑ TEER, ↓ PP of FITC-D	MID	[81]
				Unchallenged organoids: ↑ TEER		
FOS Frutalose (OFP; Sensus), DP ≤ 10	100 mg/L	PMA-exposed T84 monolayers	In vitro	Pre-incubation: ↑ TEER	MID	[179]
FOS Orafti^®^ L95, 75% *w*/*w* syrup, FOS:94.8 *w*/*w* ds	2% *v*/*v*	Caco-2 monolayers	In vitro	↑ TEER, no effect on TJ gene or mRNA expression	MID	[171]
FOS (Orafti^®^, Beneo Orafti, Tienen, Belgium) P95, DP 7–8	2%	DON-exposed Caco-2 monolayers	In vitro	Apical & basolateral pre-treatment: ↑ TEER, ↓ PP of LY dose-dependently	MID	[23]
				Total treatment: ↑ TEER during calcium-switch assay at highest C		
FOS (Nutraflora^®^), DP 2–9	10% *w*/*v*	Caco-2Bbe1 monolayers	In vitro	Pre-incubation/challenged cells: unsuccessful attenuation of F-actin microfilaments rearrangement, no effect on TEER, PP of FITC-D and ZO-1 redistribution	MID	[180]
				Unchallenged cells: no effect on FITC-D PP		
FOS (34% 1-kestose, 53% nystose, 9% 1F-β-fructofuranosylnystose)	100 mmol/L	Caco-2 monolayers	In vitro	↓ TEER, ↑ PP of LY	MID	[175]
FOS (Meioligo W, Meiji Co., Tokyo, Japan)	5%	MCD mice (NASH)	In vivo	Improvement of ZO-1 abundance	MD	[184]
FOS (Nutraflora^®^)	6%	5′FU-exposed mice (Mucositis)	In vivo	Pretreatment and total treatment: ↑ mRNA ZO-1 & occludin, ↓ PP of 99mTc-DTPA	MD	[185]
FOS (Nutraflora^®^)	6%	5′FU-exposed mice (Mucositis)	In vivo	↓ PP of 99mTc-DTPA	MD	[186]
FOS (P95S, Quantum Hi-Tech Biological Co. Ltd., Guangdong, China)	1.2%	Chronic stress exposed mice	In vivo	↑ mRNA & proteins Claudin-1, Occludin & ZO-1	MD	[187]
FOS	4 g/kg/day	HFD-fed mice (NAFLD)	In vivo	FOS alone and as synbiotic (*Lactobacillus paracasei* N111): ↑ occludin-1 & claudin-1 proteins	MD	[188]
FOS (Solarbio Biotechnology, Beijing, China), 95.93%	2%/day	OVA-exposed mice (Food allergy)	In vivo	Enhancement of TJ complex- electron density	MD	[189]
PB (Center for Anti-aging Research, Nu Skin Enterprises, Shanghai, China) composed of GOS, FOS, inulin, and anthocyanins	1.26 mg/g/day	*Trichinella spiralis-*exposed mice (IBS)	In vivo	Pretreatment and total treatment: ↑ occludin	MD	[163]
FOS (Nutraflora^®^), DP 2–9	10% *w*/*v*	*Citrobacter rodentium*-exposed mice	In vivo	No effect on FITC-D PP	MD	[180]
FOS (Meiji Seika Kaisha, Ltd., Tokyo, Japan), 6.5% GF5, 43.4% GF4, 40.9% GF3, 7.1% 1-kestose GF2, 2.1% glucose and fructose.	4 g/kg/day	Healthy weaned piglets	In vivo	↑ mRNA ZO-1, occludin & claudin-1	MD	[170]
Shanghai Lanpu Biotechnology Co., Ltd., Shanghai, China; FOS ≥ 20%	2.5 mg/kg/day	ETEC-exposed weaned piglets	In vivo	↑ mRNA ZO-1 & occludin (exceeding control)	MD	[190]
FOS, GOS, MFGM	7.5 g/L/day	Weaned piglets	In vivo	↑ mRNA ZO-1, claudin-1, occludin & E-cadherin	MD	[191]
FOS purity 93%g; Raftilose P95, Orafti^®^	60 g/kg/day	*Salmonella enterica*-exposed rats	In vivo	↑ urinary CrEDTA excretion (through TJ)	MD	[195]
FOS purity 93%g; Raftilose P95, Orafti^®^	60 g/kg/day	Healthy rats	In vivo	↑ urinary CrEDTA excretion (through TJ), no alterations on cadherins, ZO-1 claudin 2 & 4 genes expression	MD	[196]
Enzymatically synthesized FOS, DP 3.5, MW 550Da	5 g/day	SHIME^®^ inoculated with fecal sample from IBD patient and coupled with co-cultures of Caco-2 cells and THP1 macrophages	In vitro	↑ TEER	MD	[192]
FOS Orafti^®^ were boiled for 20min, following in vitro digestion and human fecal fermentation	50 mg of an equivalent carbohydrate was fermented using 5% of fecal inoculum	Caco-2 cells incubated with FOS ferment supernatant	In vitro	↑ TEER	MD	[193]
FOS (Sigma-Aldrich), chicory root-originated, ≥90%	5g/L	SHIME^®^ inoculated with fecal sample from healthy donors and coupled with co-cultures of Caco2:HT29-MTX-E12	In vitro	↑ TEER	MD	[194]

99mTc-DTPA, Technetium-99mTc-DTPA; DON, Mycotoxin deoxynivalenol; EHEC, Enterohemorrhagic *E. coli*; ETEC, Enteropathogenic *E. coli*; FITC-D, Fluorescein isothiocyanate–dextran; FOS, Fructooligosaccharides; FU, Fluorouracil; GOS, Galactooligosaccharides; LY, Lucifer yellow; IBS, Irritable bowel syndrome; MCD, Methionine-choline-deficient; MD, Microbiota-dependent; MID, Microbiota-independent; NASH, Non-alcoholic steatohepatitis; OVA, Ovalbumin; PMA, Phorbol 12-myristate 13-acetate; PP, Paracellular permeability; SHIME, Simulator of the Human Intestinal Microbial Ecosystem; TEER, Transepithelial electrical resistance; TJ, Tight junction.

### 4.2. Galactooligosaccharides

#### 4.2.1. Structure and Sources

**Figure 4:**
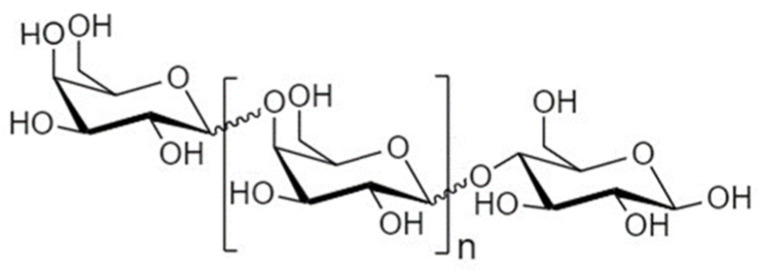
GOS structure: the main components of GOS are n 1,4- and 1,6-linked β-galactose monomers and a terminal β-d-galactose or β-d-glucose. GOS, galactooligosaccharides.

Like FOS, the galactooligosaccharides (GOS) are considered typical prebiotics [199], which are extensively added in infant formulas due to their structural and functional resemblance to oligosaccharides present in human breast milk [165,200]. Even though the natural source of GOS is mammalian milk [201] and galactose constitutes a principal component of some human milk oligosaccharides (HMOs), GOS have distinct structures compared to HMOs [161]. GOS are carbohydrates based on the milk sugar lactose, consisting of 1–7 galactose moieties with a terminal gal or glucose monomer and a DP ranging from 2–8 [23,202,203] (Figure 4). The production of GOS occurs upon β-galactosidase-mediated transgalactosylation of lactose, resulting in heterogeneous mixtures of oligomers linked via β-(1→2, 3, 4, or 6) glycosidic bonds, while in nature, most of these linkages are 1→4 and 1→6 oriented [161,162,165]. Notably, the vast majority of studies have implemented beta-linked GOS, whilst the less studied alpha-linked forms also occur [199]. Nonetheless, the nature of the linkages present in the examined GOS mixtures is not always clarified [161], halting the establishment of structure–activity relationships.

#### 4.2.2. Microbiota-Independent Effects on TJs

##### *GOS* *Reinforce the Integrity of TJ under Normal and Pathological Conditions*

Numerous in vitro studies have established that GOS directly interact with IECs and enhance TJ dynamics (Table 2). GOS not only attenuate TJ impairments but also seal the paracellular space of IECs under normal conditions [23,171,204,205,206,207]. The microbiota-independent barrier protective effects of GOS are influenced by several factors. First, based on studies using Caco-2 cells, GOS pre-treatment reduces the increased paracellular permeability caused by DON and heat stress challenges and promotes TJ re-assembly during a calcium switch assay in a concentration-dependent manner [23,205]. The acceleration of the TJ assembly in Caco-2 cells is also time-dependent, indicating that the period of treatment exposure is of key importance [23,204]. In addition, the barrier-protective effects are not evident when the monolayers are co-incubated with GOS and DON, indicating the significance of treatment timing [204]. Regarding structure–activity relationships, lower DP (i.e., DP 2) seem to be more potent regarding their barrier protective effects than higher DP (i.e., DP 3) [23]. Consequently, the potential of GOS formulations to strengthen the paracellular fence is highly dependent on the physicochemical properties of the compounds present, as determined by their DP and the overall composition of the mixture [23]. Intriguingly, GOS better prevent the loss of barrier function when applied both apically and basolaterally versus solely apically [204], probably stressing the significance of cell surface receptors located on both sides of the IECs.

##### *Postulated* *Underlying Mechanisms*

Until now, an explicit mechanism explaining the GOS-mediated enhancement of TJ function has not been identified. Based on a recent study by Lafontaine et al., exposure of Caco-2 cells to GOS enhances the integrity of the monolayer without affecting the TJ gene or protein expression [171]. By contrast, genes related to metabolic processes and cell membrane transport are upregulated, suggesting that GOS induce re-arrangements of the sterol/fatty acids and collagen of the cell membrane and alter the energy-dependent transmembrane trafficking of solutes. These compositional changes, rather than any interference with the genome or transcriptome, may result in the observed TEER increase [171]. Even though the exact ways GOS promote TJ dynamics remain elusive, an indisputable mechanism implicates their immunomodulatory effects. GOS have been suggested to directly interact with cell receptors, namely TLR4, peptidoglycan recognition protein 3 (PGlyRP3), PPARγ, and C-type lectin receptors on IECs as well as on immune cells such as DCs [171,182,207,208,209]. Indeed, GOS individually or in mixtures exert anti-inflammatory effects on intestinal epithelial monolayers in vitro and suppress the expression and secretion of inflammatory cytokines such as IL-8, IL-1β, and INF-γ and halt TLR4/NF-κΒ/TNF-α responses [23,204,206,210,211]. Nonetheless, differences in the GOS-mediated immunomodulatory effects are observed based on the in vitro model studied, probably due to differentially expressed receptors among the various cell lines, e.g., low mRNA TLR4 expression in Caco-2 cells [207,208]. Furthermore, in co-cultures of IECs with DCs, GOS have been shown to decrease IL-12, IL-6, IL-8, and increase the ratio IL-10/IL-12, in a TLR/MyD88 dependent manner [209]. Collectively, these data indicate that the intact GOS molecules protect the intestinal epithelial barrier, at least in part, via regulation of anti- and pro-inflammatory responses known for interfering with TJ functionality. In addition, GOS promote cell differentiation and re-epithelialization in vitro, an effect suggested to be responsible for the observed decrease in paracellular permeability [207,212]. Further studies are warranted to shed light on the complex underlying mechanisms contributing to the microbiota-independent ability of GOS to promote TJ functionality.

#### 4.2.3. Microbiota-Dependent Effects on TJ

The prebiotic-related barrier protective effects of GOS have been explored in quite a few in vivo studies individually [200,202,203,204,213,214,215,216,217] and as components of more complex dietary blends [163,191,218]. Not surprisingly, considering the structural resemblance to HMOs, GOS exert numerous beneficial effects in animal models, including the protection and reinforcement of the intestinal epithelial barrier (Table 2). Indeed, GOS supplementation of ETEC- [202], LPS- [214], and DON-treated mice [204], heat stress-treated broilers [213], and severe acute pancreatitis (SAP)-rats [216] mitigates disturbances in TJ abundance and localization, maintaining the epithelial integrity. In addition, GOS have been shown to modulate TJ expression on a transcriptional and protein level in both the small and large intestines of healthy suckling and weaned piglets [200,203,215,217]. These findings indicate that GOS supplementation may seal the paracellular route even under non-stress conditions. On the other hand, Barrat et al. observed that GOS/Inulin (8:12)-supplementation in neonatal rats increases bacterial translocation [218]. However, no alterations in gut permeability or the levels of the pore-forming claudin-2 and claudin-3 mRNAs are observed. The impaired barrier function can be attributed to the increased production of the organic acids acetate and lactate, yet the exact underlying mechanism remains to be elucidated [218]. The ability of GOS to modulate TJ abundance and organization in vivo is mainly ascribed to their prebiotic activity. GOS promote the growth of health-associated commensals, such as *Bifidobacterium* spp., *Lactobacillus* spp., *A. muciniphila,* and *Ruminococcus* spp., increase the Bacteroides/Firmicutes ratio, elevated butyrate, acetate, propionate, and pentanoate levels, and restore dysbiotic populations with parallel suppression of inflammatory activity [200,202,203,214,215,216,217,219,220,221]. The GOS-induced changes in microbial communities and SCFA levels are highly correlated to the changes in TJ expression and inflammation markers in the abovementioned studies, verifying that the barrier-protective effects of GOS are microbiota-dependent. Indeed, a study by Wang et al. demonstrates increased AMPK stimulation and suppressed inflammatory-NF-κΒ signaling after treatment of piglets with GOS; a finding attributed to the ability of SCFAs to promote TJ formation and exert anti-inflammatory effects [200].

**Table 2:** Microbiota-dependent and independent effects of GOS on paracellular permeability and/or TJs.

Treatment Characteristics	[GOS]	Model/Experimental Setup	Type of Study	Observed Effects on PP and/or TJs	Type of Effect	References
Vivinal^®^ GOS syrup (FrieslandCampina Domo, The Netherlands), 45% GOS, DP 2–8	2%	DON-exposed Caco-2 monolayers	In vitro	↑ TEER (Acceleration of TJ reassembly) during Ca^2+^ switch, Pre-incubation: ↑ TEER, ↓ PP of LY, FITC-dextran, attenuation of claudin-3 disturbed expression and localization	MID	[204]
Vivinal^®^ GOS syrup, (FrieslandCampina Domo, The Netherlands), 59% GOS, DP 2–6	0.5, 1, 2% *w*/*w*	DON-exposed Caco-2 monolayers	In vitro	↑ TEER during Ca^2+^ switch (2% time dependently), Pre-incubation: ↑ TEER, ↓ PP of LY dose dependently	MID	[23]
Individual DP2, DP3 from Vivinal^®^ GOS syrup	0.75%			↑ TEER (both), ↓ PP of LY (only DP2), combination: ↑ TEER, ↓ PP of LY		
Purified Vivinal^®^ GOS, (FrieslandCampina Domo, The Netherlands), 97% GOS	2% *w*/*v*			↑ TEER during Ca^2+^ switch, Pre-incubation: ↑ TEER, ↓ PP of LY		
Vivinal^®^ GOS syrup (FrieslandCampinaDomo, The Netherlands), DP 2–8, 59% *w*/*w* GOS	1, 2.5%	Heat stress-exposed Caco-2 monolayers	In vitro	Pre-treatment: ↑ TEER, ↓ PP of LY	MID	[205]
Nutrabiotic^®^ GOS (Dairy Crest Ltd., Esher, Surrey, UK), 66.5% *w*/*w* dry solids GOS, DP 2–7	2% *v*/*v* = 1.4% *w*/*v* GOS	Caco-2 monolayers	In vitro	↑ TEER after 24 h-exposure, no effect on TJ gene or mRNA expression	MID	[171]
Vivinal^®^ GOS-WPC (FrieslandCampina, Amersfoort, The Netherlands), 27.5% GOS	100 μg/mL	Caco-2 monolayers	In vitro	Non-significant increase in TJ mRNA/protein levels	MID	[206]
		HT-29-MTX monolayers		↑ claudin-1, occludin, ZO-1 mRNA, ↑ claudin-1, -3, occludin, ZO-1 proteins		
		TNF-α-exposed Caco-2/HT-29-MTX co-culture monolayers		Pre-treatment: ↑ TEER		
Vivinal^®^ GOS syrup (FrieslandCampina) with GOS- plant sterol enriched milk-based fruit beverages (Global Technology Center, Alcantarilla, Murcia, Spain), 1.8 g/100 mL	1:5 *v*/*v*	Caco-2 monolayers	In vitro	↑ TEER under oxidative stress-challenged and unchallenged conditions	MID	[207]
Vivinal^®^ GOS syrup (FrieslandCampina Domo, The Netherlands), 45% GOS, DP 2–8	1% GOS (1 kg containing 22.22 g/kg VGOS)	DON-exposed challenged mice	In vivo	Prevention of claudin-3 mRNA overexpression and maintenance of its cellular distribution, ↓ claudin-2 mRNA without attenuation of FITC-D permeability	MD	[204]
Yuanye Biotechnology Co. (Shanghai, China)	0.2 g/100 g BW	*E. coli* O157-exposed mice	In vivo	↑ mRNA occludin, claudin, ZO-1	MD	[202]
GOS 100%: 52.86% trisaccharide, 36.39% tetrasaccharide, 10.75% oligosaccharides with DP ≥ 5 (patent application no. 202011427659.7, China)	0.25, 0.5g/kg BW	LPS-exposed mice	In vivo	Pre-treatment: ↑ mRNA ZO-1, occludin, claudin-1	MD	[214]
Vivinal^®^ GOS syrup (FrieslandCampin Domo, Borculo, The Netherlands), DP 2–8, 59% *w*/*w* GOS	1, 2.5%	Heat stress-exposed broilers	In vivo	Total treatment: attenuation of ↑ mRNA expression of claudin-5 and ZO-1 dose dependently	MD	[213]
GOS-90S Quantum Hi-Tech Biological Co., Ltd. (China), DP 2–8, 90% (*w*/*w*) GOS	1 g/kg BW/day	Suckling piglets	In vivo	↑ occludin mRNA, ↑ ZO-1, occludin protein levels	MD	[203]
Quantum Hi-Tech Biological Co., Ltd. (China), 90% GOS	1 g/kg BW/day	Suckling piglets	In vivo	↑ ZO-1, claudin-1 but not occludin protein levels	MD	[200]
Vivinal^®^ GOS syrup 75% DM (FrieslandCampinaDomo, Borculo, The Netherlands), DP 2–8, 59% *w*/*w* GOS	600–1600 mL/piglet/day (0.8% FOS)	Weaned piglets	In vivo	↑ claudin-1, ZO-2 mRNA and ZO-1, occludin mRNA and protein levels	MD	[217]
GOS/CGMP 2:1 (Beijing Sanyuan Foods Co., Ltd., Beijing, China), 90% GOS *w*/*w*, DM 3	10 g/day	Piglets fed by GOS treated sows	In vivo	↑ mRNA claudin-1, claudin-2, occludin	MD	[215]
Xi’an Deshipu Bio-industry Company, China, 90% GOS	1 g/day	Sodium taurocholate-exposed rats (SAP)	In vivo	↑ occludin mRNA and protein (not significant but positively correlated to mRNA increase)	MD	[216]
Vivinal^®^ GOS (Friesland Foods Domo, Zwolle, The Netherlands) i.e., 4.9 g/L GOS/Inulin (Beneo HP, Orafti, France) i.e., 0.70 g/L Inulin, (88/12, 5.6 g/L)	10.9 g/L/day (V dependent of age)	Newborn rats	In vivo	No alterations in mRNA claudin-2, -3, no effect on PP of Dextran, ↓ mRNA ZO-1	MD	[218]

DON, Mycotoxin deoxynivalenol; FITC-D, Fluorescein isothiocyanate–dextran; GOS, Galactooligosaccharides; LY, Lucifer yellow; MD, Microbiota-dependent; MID, Microbiota-independent; PP, Paracellular permeability; SAP, Severe acute pancreatitis; TEER, Transepithelial electrical resistance; TJ, Tight junction.

### 4.3. Alginate Oligosaccharides

#### 4.3.1. Structure and Sources

**Figure 5:**
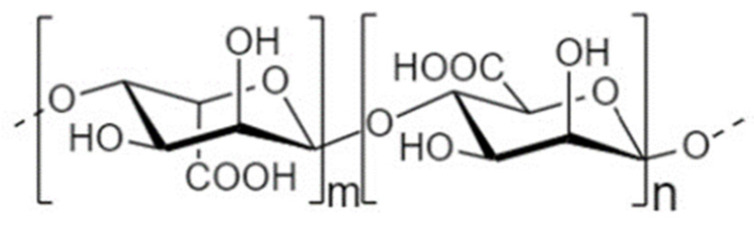
AOS structure: the main components of AOS are 1,4-linked β-d-mannuronic acid and 1,4-linked α-l-guluronic acid. AOS, alginate-oligosaccharides.

Alginate Oligosaccharides (AOS) have attracted tremendous attention over the past years due to their versatile properties, including immunoregulatory, anti-inflammatory, anti-apoptotic, antibacterial, neuroprotective, antihypertensive, anti-coagulant, anti-tumor, anti-oxidative, hypolipidemic, and hypoglycemic activities [222,223,224]. In addition, AOS have emerged as promising prebiotic agents with favorable physicochemical characteristics for food applications, yet their effects on gut flora regulation have just started to be explored [225,226]. AOS are acidic anionic carbohydrates obtained upon acid hydrolysis, enzymatic or oxidative degradation, as well as microbial fermentation of alginate, a biopolymer isolated from the cell walls of brown algae, i.e., seaweed [165,222,227]. AOS are low molecular weight linear polymers that contain 2–25 monomers and are composed of two types of uronic acid that are conformational isomer residues: (1→4)-linked-α-L-guluronic acid (GulA/G) and (1→4)-linked-β-d-mannuronic acid (Man/M) [222] (Figure 5). These monomers form either homooligomeric blocks (GG, G-blocks/MM, M-blocks) or hetero-oligomeric mixed sequences (G-M, GM-blocks) [165,226,228]. Interestingly, the structural characteristics of AOS, i.e., spatial conformation, MW, G content (M/G ratio), and MG sequence, have a substantial impact on biological activity [224]. Depending on the depolymerization process, various complex mixtures of AOS with unknown characteristics are obtained, hence AOS of similar DP may exhibit different functionalities [222].

#### 4.3.2. Microbiota-Independent Effects on TJs

##### *AOS* *Interact with the Mannose Receptor and Reinforce the Intestinal Epithelial Barrier*

Currently, limited data is available regarding the barrier-protective effects of intact AOS on the IECs. Nonetheless, the interest in the underlying mechanisms has grown over the past two years, shedding light on the signaling pathways involved. Based on studies using porcine intestinal monolayers, AOS directly reinforce the mechanical barrier by increasing the abundance of TJs at protein and transcriptional level under both normal and challenged conditions (Table 3). Recently, Zhang et al. identified the involvement of the mannose receptor (MR) signaling in the AOS-mediated barrier-protective effects [229]. The MR is a carbohydrate-binding receptor of the C-type lectin superfamily that recognizes mannose, fucose, or *N*-acetyl-d-glucosamine (GlcNAc) residues. Even though considered an intracellular receptor, it is constantly recycled between the plasma membrane and the endosomal apparatus, recognizing a wide range of endogenous and exogenous ligands. MR is involved in various cellular processes, namely innate immune activation, production of pro- and anti-inflammatory cytokines, homeostatic processes, and recognition of pathogens, through interactions with other canonical PRRs such as the TLR2 [230,231,232]. According to the latest studies, AOS seem to bind to the MRs of the IECs, leading to increased expression of transcriptional factors, resulting, among others, in improved cell junction formation [229,233].

##### *AOS* *Rescue the Intestinal Epithelial Integrity via Their Anti-Inflammatory and Anti-Apoptotic Properties*

Based on two recent studies on the IPEC/J2 cell line, AOS prevent the inflammatory injury induced by stimuli such as TNF-α and LPS, with subsequent attenuation of disturbances on TJ mRNA and protein levels. Indeed, pre-treatment of the monolayers with AOS alleviates the TNF-α-mediated IL-6 and TNF-α release, saving occludin’s expression and abundance. The rescue of TJ integrity is, at least in part, attributed to the TNF Receptor 1 (TNFR1)-dependent anti-apoptotic effects of AOS [227]. Similarly, AOS pre-treatment reduces the LPS-induced production of TNF-α and INF-γ and suppresses cell apoptosis, preserving occludin’s abundance in vitro and in vivo in ETEC-infected pigs. In both cases, AOS seem to competitively inhibit the binding of LPS to the cell surface receptors, such as TLR4, subsequently repressing NF-κΒ p65-mediated downstream inflammatory cascades [228]. These results agree with another study on HT-29 cells, demonstrating that AOS pretreatment attenuates *E. coli*-induced IL-8 release, establishing this oligosaccharide as a potent anti-inflammatory agent capable of protecting the integrity of intestinal TJs [234].

#### 4.3.3. Microbiota-Dependent Effects on TJs

Like other NDOs, apart from strengthening the intestinal barrier as intact compounds, AOS can protect TJ function via their prebiotic-related activities. Based on quite a few in vivo studies, AOS promote health-beneficial bacteria populations, e.g., Bacteroidetes and Firmicutes, and diminish the numbers of harmful bacteria such as Enterobacteriaceae. Consecutively, the elevated levels of SCFA, majorly acetate, butyrate, and propionate, exert numerous beneficial effects on the host, including immunomodulation and protection of the intestinal barrier’s integrity (Table 3) [226,235,236,237]. A study by Wan et al. showed that AOS supplementation in weaned pigs promotes TJ gene expression, an effect initially attributed to the AOS-mediated accelerated intestinal growth and development [238]. However, a later study by the same group demonstrated that the reinforcement of the intestinal barrier is related to increased SCFA levels. Indeed, AOS increase p-AMPK levels and decrease the ΝF-κB p65 abundance, clearly improving TJ functionality [225]. In addition, these findings agree with studies on dextran sulfate sodium (DSS)-challenged [239] and HFD-fed [240] mice, as well as in mucositis-challenged mice treated with AOS originated-fecal microbiota transplants [241]. Not surprisingly, unsaturated AOS (UAOS) and AOS supplementation alleviates inflammatory responses and restores dysbiotic populations, thus rescuing TJ protein abundance and conferring overall protection to the intestinal barrier of the mice.

**Table 3:** Microbiota-dependent and independent effects of AOS on paracellular permeability and/or TJs.

Treatment Characteristics	[AOS]	Model/Experimental Setup	Type of Study	Observed Effects on PP and/or TJs	Type of Effect	References
AOS (Qingdao Bozhihuili Co., Ltd., Qingdao, China)	10 μg/mL, 100 μg/mL	IPEC/J2 monolayers	In vitro	↑ claudin protein levels	MID	[229]
AOS (Qingdao Bozhihuili Co., Ltd.)	10 μg/mL, 100 μg/mL	IPEC/J2 monolayers	In vitro	↑ TEER time dependently, ↑ claudin, occludin protein levels dose dependently	MID	[233]
AOS prepared by depolymerization of alginate (Qingdao Bright Moon Seaweed Group Co., Ltd., Qingdao, China)	600 μg/mL	TNF-α-exposed IPEC/J2 monolayers	In vitro	↑ occludin mRNA and protein levels and ↑ occludin protein levels of unchallenged cells	MID	[227]
AOS prepared by depolymerization of alginate (Qingdao Bright Moon Seaweed Group Co., Ltd., Qingdao, China), DP 2–8	600 μg/mL	LPS-exposed IPEC/J2 mono-layers	In vitro	↑ occludin protein abundance	MID	[228]
AOS prepared by depolymerization of alginate (Qingdao Bright Moon Seaweed Group Co., Ltd., Qingdao, China), DP 2–8	10 mg/kg BW	ETEC-exposed weaned pigs	In vivo	↑ occludin protein abundance	MID	[228]
ALGO (Dalian Institute of Chemical Physics, Chinese Academy of Sciences, Dalian, China), DP 4.4	100 mg/kg BW	Weaned pigs	In vivo	↑ occludin, ZO-1 mRNA	MD	[238]
AOS (-)	100 mg/kg BW	Weaned pigs	In vivo	↑ occludin, claudin-1 mRNA, no alteration for ZO-1, 2 mRNA	MD	[225]
UAOS prepared according to Li et al. [242], MW 420.4	400 mg/kg BW/day	HFD-fed mice	In vivo	↑ ZO-1, occludin protein abundance	MD	[240]
UAOS prepared according to Li et al. [242]	200, 400 mg/kg BW/day	DSS-exposed mice (UC)	In vivo	↑ ZO-1, occludin protein abundance dose dependently	MD	[239]
AOS (-)	10, 100 mg/kg BW	FMT (fecal microbiota transplantation)-treated mice exposed to busulfan (mucositis)	In vivo	↑ ZO-1, claudin protein abundance, ↑ occludin protein levels	MD	[241]

AOS, Alginate oligosaccharides; DSS, Dextran sulfate sodium; ETEC, Enteropathogenic *E. coli*; FITC-D, Fluorescein isothiocyanate–dextran; HFD, High-fat diet; MD, Microbiota-dependent; MID, Microbiota-independent; PP, Paracellular permeability; TJ, Tight junction; UAOS, Unsaturated AOS; UC, Ulcerative colitis.

### 4.4. Chitooligosaccharides

#### 4.4.1. Structure and Sources

**Figure 6:**
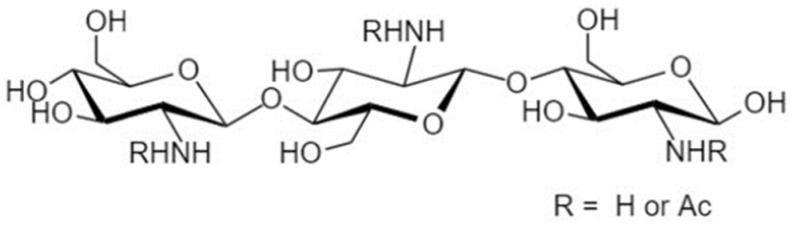
COS structure: the main components of COS are 1,4-linked GlcNAc and 1,4-linked GlcN. COS, chitin/chitosan-oligosaccharides.

Chitin- or chitosan-oligosaccharides (COS) are among the most exhaustively investigated NDOs with a wide range of potential applications in pharmaceutical, biomedical, nutritional, cosmeceutical, agricultural, and environmental fields due to their innumerable functional properties [161,243,244]. Of tremendous interest are the diverse biological functionalities exerted by COS and their derivatives, such as anti-inflammatory, anti-tumor, anti-microbial, anti-oxidant, anti-hypertensive, anti-diabetic, anti-HIV-1, anti-Alzheimer, hypocholesterolemic, immunostimulatory, prebiotic, tissue regenerative, hemostatic, calcium-absorption enhancing, and drug/DNA delivery abilities [243,245,246,247]. COS are prepared from the deacetylation and degradation of chitin or depolymerization of chitosan via chemical, enzymatic processes, or both [245,246,248]. These are linear polymers consisting of β-(1→4)-linked *N*-acetyl-2-amino-2-deoxyglucose (*N*-acetyl-d-glucosamine/GlcNAc, acetylated unit A) and β-(1→4)-linked d-glucosamine (GlcN, deacetylated unit D) [244,245,247] (Figure 6). Chitin constitutes the second most abundant polymer in nature [246], found in the exoskeletons of arthropods and crustaceans and the cell walls of fungi, algae, and yeast with a high proportion of GlcNAc (Degree of acetylation (DA) > 70%). By contrast, chitosan, which is mainly composed of GlcN (DA < 30%), is rarer and can be extracted from the cell walls of specific fungi [161,165,245]. COS are oligomers with an average MW < 3.9 kDa (though not strictly), DP < 20, and a high degree of deacetylation, DD > 90% (DD: the molar units of GlcN in the COS backbone) [165,244,245,246]. Notably, the chemical characteristics of COS mixtures, i.e., MW distribution (polydispersity or PD), DP, DD, and the sequence or pattern of N-acetylation (PA)/charge distribution, have a tremendous impact on the exhibited physicochemical and ultimately biological properties and vary depending on the preparation method followed and staring material [245,246].

#### 4.4.2. Microbiota-Independent Effects on TJs

##### *COS* *Facilitate TJ Re-Assembly via AMPK Stimulation and Promote TJ Integrity*

COS can be readily absorbed through the intestinal epithelium upon ingestion, on which they exert direct effects, and then they enter the bloodstream to induce their health-promoting systemic activities [246,247,249,250]. Among their beneficial properties, COS reinforce and protect the integrity of the intestinal epithelial barrier (Table 4). A study on T84 monolayers by Muanprasat et al. revealed that COS accelerate TJ re-assembly upon activation of CaMKKβ/AMPK pathway similar to FOS [251]. Specifically, COS, which are positively charged under physiological pH, directly interact with the CaSR of IECs, leading to the release of [Ca^2+^]i from the ER and mitochondria, at least in part, via the CaSR-Gq-PLC-IP3-receptor channel-dependent cascade, ultimately leading to AMPK stimulation and TJ re-assembly [251]. This effect is both concentration- and MW-dependent; the low MW (LMW) = 5 kDa COS is more potent than the high MW COS (HMW = 8 and 14 kDa) [251]. This is in agreement with other studies establishing LMW COS as more promising with regard to biological activities [252]. Of note, the COS-mediated AMPK activation is not cell-line specific as pAMPK is found in Caco-2 and HT-29 monolayers upon incubation with COS, though the effect is observed in higher concentrations and after a more extended time frame (1 h for T84 vs. 24 h for Caco-2 and HT-29 cells) [251]. Accordingly, 24-h incubation of IPEC-J2 cells with COS increases TEER following a concentration-dependent trend [249]. By contrast, treatment of Caco-2 cells with ascending COS concentrations with DD > 70% fails to induce any changes in TEER or [^14^C] mannitol permeability [253]. Notably, COS also exert reparatory effects on TJs. Post-DSS challenge-treatment of Caco-2 cells with LMW COS restores occludin mRNA expression and abundance, while HMW COS induce a slighter effect [254]. Collectively, these results emphasize the significant dependency of the observed outcome on the concentration, incubation period, and physicochemical COS characteristics, as well as the importance of the cell line used in the different studies [249,251,254].

##### *COS* *Alleviate Inflammation and Regulate TJ Abundance*

The anti-inflammatory properties of COS are well documented, including studies focusing on the impacts on IECs. Studies on TNF-α- and LPS-challenged T84 monolayers have demonstrated that COS inhibit the TLR4/NF-κB signaling and subsequent inflammatory responses, protecting the intestinal epithelial barrier, based on TEER and FITC-D permeability assays [248,251]. Even though AMPK is a known negative regulator of NF-κB, its activation is not responsible for the elicited anti-inflammatory effect against LPS in T84 monolayers [251]. However, the CaSR seems to be implicated in the anti-inflammatory activity since COS administration in LPS-challenged piglets inhibits the TLR4/NF-κΒ inflammatory cascade with a parallel increase in CaSR expression in both challenged and healthy piglets [255]. COS pre-incubation of LPS-challenged IPEC-J2 monolayers attenuates barrier impairments with parallel suppression of inflammatory responses via TLR4/NF-κΒ signaling. Strikingly, even though the maximal barrier-protective effect occurs in a concentration-dependent manner (maximal effect with 800 μg/mL), the best anti-inflammatory activity is exerted by the lowest concentration (200 μg/mL). Whether the CaSR signaling is involved in the barrier-protective effects was not investigated in this study [249]. Furthermore, based on another study implementing TNF-α-challenged IPEC-J2 cells, co-incubation with LMW COS attenuates the upregulation of claudin-1 mRNA induced by TNF-α, and tends to decrease ZO-1 mRNA. Considering that these transcriptional changes are not accompanied by a TEER drop, COS do not seem to compromise TJ integrity but rather to mitigate TNF-α-mediated changes in TJ gene expression. Concurrently, COS suppress the overexpression of inflammatory cytokines, at least in part, via the cAMP/PKA pathway, which inhibits the translocation of NF-κB into the nucleus, while this effect is MR-independent [256].

#### 4.4.3. Microbiota-Dependent Effects on TJs

The prebiotic activity of COS has been extensively studied in vivo, in weaning piglets [257,258,259], mini-piglets [260], broilers [261], mice [262], rats [263,264], and in in vitro fermentation models [262,265,266]. Like every other bio-functionality of COS, their effect on the growth of various microbial species is closely related to the average MW and DD of the investigated COS mixture. These factors, along with the different experimental settings, may explain contradictory results found in the literature [262,266]. For instance, COS promote the growth of health-beneficial species such as *Lactobacillus* spp., *Bifidobacterium* spp., *Akkermansia* spp., and *Parabacteroides* spp. while reducing the numbers of potential bacterial pathogens, including *E. coli*, *B. fragilis*, *Streptococcus* spp., *Enterococcus* spp., *Clostridium* spp., and the *Proteobacteria* phylum, with a parallel elevation of acetate, butyrate, propionate, valerate, and total SCFA levels [257,260,261,267,268,269]. By contrast, other studies have demonstrated that COS may reduce or not affect the Bifidobacterial, and Lactobacilli counts and elevate pathogenic populations such as *Escherichia* spp. [259,262,263,264].

Over the past few years, numerous studies established the prebiotic properties of COS and linked their ability to restore dysbiotic populations with the barrier-protective effects observed (Table 4). Indeed, chito- and chitosan-oligosaccharides restore or significantly ameliorate the balance between beneficial and inflammogenic microbes in weaned piglets [267], mice models of metabolic syndrome/obesity [269,270], NAFLD [271], diabetes [268], SAP [272] and loperamide-induced constipation [273], with the parallel recovery of reduced TJ protein and mRNA levels and repression of inflammation, as a result of the elevated SCFA production. Supplementation of diabetic mice with COS restores occludin abundance with parallel suppression of p38 and elevation of AMPK, consistent with the fact that stimulation of p38 and AMPK leads to gut barrier disruption and tightening, respectively [268]. Interestingly, according to Spearman correlation analyses, COS supplementation favors and suppresses the growth of bacteria related to the TJ proteins and inflammation, such as the *Bifidobacterium* and *Bosea* genera, respectively [268,270]. Moreover, supplementation of HFD-fed mice with COS of an average MW 879.6 Da exerts a better effect than COS of an average MW 360.9 Da regarding the stimulation of the barrier’s integrity, emphasizing the significance of the structure-activity relationship [269]. In addition, studies on healthy broilers [274] and DSS-challenged mice [254] have also established COS as an intestinal barrier-protective agent, even though the correlation between the alterations in TJ abundance and microbial communities’ structure was not investigated. Contrary to the above, supplementation of weaned pigs with a low COS dosage compromised the intestinal barrier integrity, as shown by the decreased TJ mRNA expression and increased serum D-lactate and diamine oxidase. This effect is accompanied by an increased immune and oxidative stress response, though this discrepancy seems to be related to the dosage and characteristics of the COS treatment. However, whether shifts in microbial populations accompanied this effect was not examined [275].

**Table 4:** Microbiota-dependent and independent effects of COS on paracellular permeability and/or TJs.

Treatment Characteristics	[COS]	Model/Experimental Setup	Type of Study	Observed effects on PP and/or TJs	Type of Effect	References
COS (Kitto Life Co., Ltd., Kyungki-do, Seoul, Korea), MW 5–10 kDa, >70% COS content, DD > 70%	0.5-4 mg/mL	Caco-2 monolayers	In vitro	No effect on TEER nor on [^14^C] mannitol flux	MID	[253]
COS prepared by enzymatic hydrolysis of shrimp shell chitosan, MW 5000 Da, DD > 90%,	20, 100, 500 μg/mL	LPS-exposed T84 monolayers	In vitro	↑ TEER (best effect with 100 μg/mL)	MID	[248]
		TNF-α-exposed T84 monolayers				
COS prepared according to [248], MW 5000 Da, DD > 90%	100 μg/mL	T84 monolayers	In vitro	↑ TEER/acceleration of TJ re-assembly during Ca^2+^ assay	MID	[251]
		TNF-α-exposed T84 monolayers		↓ FITC-D flux		
COS (Beijing Zhong Tai He technology (ZTH tech, Beijing, China), MW < 1000 Da, DD > 90%, DP 2–7	50–100 μg/mL	TNF-γα-exposed IPEC-J2 monolayers	In vitro	Suppression of ↑ claudin-1 mRNA, tendency to ↓ ZO-1 mRNA concentration-dependently, no effect on TEER	MID	[256]
COS (Zhong Tai He Technology (Beijing, China), MW < 1000, DP 2–7, DD > 90%	800 μg/mL	IPEC-J2 monolayers	In vitro	↑ TEER concentration-dependently and ↓ FITC-D flux dose-dependently	MID	[249]
		LPS-exposed IPEC-J2 monolayers				
COS (GlycoBio (GlycoBio, Dalian, China), MW 363-1329 Da, DD > 95% HWCOS (Sigma (St. Louis, MO, USA), MW 4000–6000 Da, DD > 90%	200 μg/mL	DSS-exposed Caco-2 monolayers	In vitro	↑ occludin protein abundance and mRNA post-challenge (HWCOS less effectively)	MID	[254]
COS (GlycoBio (GlycoBio, Dalian, China), MW 363–1329 Da, DD > 95%	200 mg/kg BW/day	DSS-exposed mice (UC)	In vivo	↑ occludin protein abundance	Not determined	[259]
NACOS prepared as described in [270], DP 2–6, DA = 97%	200 mg/kg BW/day (1 mg/mL NACOS)	HFD-fed mice (Metabolic syndrome)	In vivo	↑ ZO-1, occludin mRNA	MD	[270]
LMW-COS enzymatically produced as described in [269], DD = 93% LMW-COS-H, MW 879.6 Da LMW-COS-W, MW 360.9 Da	400 mg/kg BW/day	HFD-fed mice (Obesity-Metabolic syndrome)	In vivo	↑ ZO-1, occludin mRNA and protein levels (LMW-COS-H)	MD	[269]
				↑ occludin mRNA and protein levels (LMW-COS-L)		
COS prepared by enzymatic hydrolysis as described by [276], DD = 88%, DP 2–6%	200 mg/kg BW/day	Loperamide-exposed mice	In vivo	↑ occludin, claudin-1 mRNA, ↑ ZO-1 and claudin-1 protein levels	MD	[273]
COS prepared as described in [268], DD = 88%, DP 2–6	200 mg/kg BW/day (1 mg/mL COS)	Lepr^db^ mutation (db/db) mice	In vivo	↑ occludin protein levels, no effect on ZO-1	MD	[268]
COS23 prepared by enzymatic degradation of COS as described in [277,278]	4% in drinking water	HFD-fed mice (NAFLD)	In vivo	↑ ZO-1, ZO-2 mRNA, tendency for ↑ occludin mRNA	MD	[271]
COS (MedChem Express, Shanghai, China), MW < 1 kDa, 91.0% COS	200 mg/kg BW/day (1 mg/mL COS)	Carulein-exposed mice (SAP)	In vivo	↑ occludin, claudin-1, no effect on ZO-1 abundance, ↓ FITC-Dextran flux	MD	[272]
COS, MW 1000–2000 Da, COS content >85%	30 mg/kg BW/day	Healthy weaned piglets	In vivo	↓ occludin and ZO-1 mRNA	Not determined	[275]
COS prepared by enzymatic hydrolysis as described in [267], DD > 95%, MW ≤ 1000 Da, DP 2–8	100 mg/kg BW/day	Healthy weaned piglets	In vivo	↑ claudin-1 and occludin mRNA (jejunum only)	MD	[267]
COS (Zhongkerongxin Biotechnology Co., Ltd., Suzhou, China), MW 1000-2000 Da, COS > 90%	30 mg/kg BW/day	Healthy broilers	In vivo	↑ claudin-3 mRNA, no alteration on occludin, claudin-2 and ZO-1 mRNA	Not deter-mined	[274]

COS, Chitosan oligosaccharides; DSS, Dextran sulfate sodium; FITC-D, Fluorescein isothiocyanate dextran; HFD, High-fat diet; MD, Microbiota-dependent; MID, Microbiota-independent; NAFLD, Non-alcoholic fatty liver disease; PP, Paracellular permeability; TEER, Transepithelial electrical resistance; TJ, Tight junction; UC, Ulcerative colitis.

### 4.5. Mannan-Oligosaccharides 

#### 4.5.1. Structure and Sources

**Figure 7:**
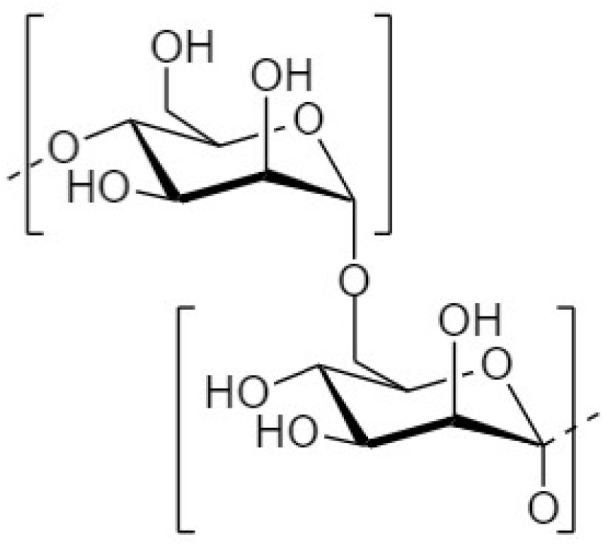
MOS structure: the main components of MOS are 1,4-linked β-d-mannose and 1,4-linked β-d-glucose or 1,6-linked α-d-galactose. MOS, mannan-oligosaccharides.

Apart from the widely studied FOS and GOS and the recently emerged COS and AOS, the mannan-oligosaccharides (MOS) are considered novel health-promoting oligosaccharides [24]. Among the beneficial properties of MOS are included prebiotic, bacterial anti-adhesive, immunomodulatory, antioxidant, antidiabetic, and anti-obesity activities [279,280,281]. MOS are functional oligosaccharides prepared mainly by hydrolysis of glucomannan or galactomannan, i.e., a mannose-consisted polymer. Konjac MOS (KMOS) are originated from glucomannan, which is abundant in the routs and tuber of *Amorphophallus* konjac (konjac) [282]. KMOS consist of β(1→4) linked β-d-glucose and β-d-mannose monomers [283]. By contrast, galactomannan-originated MOS are composed of repetitive β-(1→4)-D-mannose units with α-(1→6)-d-galactose monomers attached to the backbone (Figure 7). Galactomannan is found in the endosperm of plant species such as the fruits of coconut trees, coconut meal, and guar gum, while another source is the yeast cell wall [280,282].

#### 4.5.2. Microbiota-Dependent and Independent Effects on TJs

MOS have been suggested to confer barrier-protective effects in vivo and in vitro (Table 5). However, not much is known regarding the signaling pathways by which MOS stimulate the paracellular space sealing as intact compounds. Recently, Muanprasat et al. revealed that a galactomannan pentasaccharide promotes the integration of TJs on T84 monolayers via the AMPK-mediated acceleration of TJ re-assembly. This effect is only observed for MOS with DP 5, while DP 4, 6, and 7 fail to promote intestinal barrier integrity. Interestingly, the optimal effect is not achieved with the highest concentration used (20 μM vs. 10 μM), implying an overactivation of the AMPK signaling pathway [282,284]. In addition, co-treatment of LPS-challenged Caco-2 cells with KMOS and the probiotic *Bacillus subtilis* suppresses inflammation and saves ZO-1 and claudin-1 gene expression, rescuing the monolayer’s integrity [283].

Apart from the in vitro studies, the protective effects MOS confer towards the epithelial barrier are established by in vivo studies. Supplementation of LPS-challenged mice with KMOS or a combination of KMOS and *B. subtilis* attenuates disturbances of claudin-1 abundance with the corresponding alleviation of inflammatory responses [283]. Furthermore, KMOS treatment alleviates TJ impairments in DSS-challenged mice, an effect mediated by the specific intercellular adhesion molecule-3-grabbing nonintegrin-related 1 (SIGNR1) signaling pathway. Indeed, SIGNR1 activation is required for the macrophage phenotype switching and the consecutive decrease in paracellular permeability that are observed [285]. Yeast-derived MOS also protect intestinal TJ integrity in heat-stress-challenged broilers [286] and ETEC-challenged weaned piglets [287]. Whether these effects are partly attributed to a beneficial shift of gut flora populations was not further investigated in these studies [283,285,286,287]. Based on another study on ETEC-challenged broilers, MOS supplementation suppresses inflammation via TLR4/NF-κB signaling and mitigates TJ injury without causing any significant alterations in gut microbiota populations [288]. Nonetheless, treatment of ETEC-challenged weaned piglets with KMOS decreases *E. coli* abundance and stimulates the growth of beneficial microbes including *Lactobacillus* spp. and *Bifidobacterium* spp., with parallel alleviation of inflammation and increase in ZO-1 and claudin-1 mRNA levels [280], indicating that MOS can protect the intestinal barrier via restoration of dysbiotic populations.

**Table 5:** Microbiota-dependent and independent effects of MOS on paracellular permeability and/or TJs.

Treatment Characteristics	[MOS]	Model/Experimental Setup	Type of Study	Observed Effects on PP and/or TJs	Type of Effect	References
MOS prepared by enzymatic hydrolysis of copra milk galactomannan, DP = 5	10, 20 μM	T84 monolayers	In vitro	↑ TEER	MID	[282,284]
	10 μM			↑ TEER/acceleration of TJ assembly during Ca^2+^ assay		
KMOS/+*B. subtilis*	2 g/L	LPS-exposed Caco-2 monolayers	In vitro	↑ ZO-1, claudin-1 mRNA	MID	[283]
KMOS/+*B. subtilis*	2 g/L	LPS-exposed mice	In vivo	↑ claudin-1	Not determined	[283]
KMOS (Xi’an Yuanseng biological technology Corporation, Xi’an, China), DP 2–6	400 mg/kg BW/day	DSS-exposed mice (UC)	In vivo	↓ FITC-D flux, ↑ ZO-1, occludin	Not determined	[285]
MOS (Sichuan Junzheng Bio. Co., Ltd.), 98.5% MOS	3 g/kg BW/day (0.3%)	ETEC-exposed weaned piglets	In vivo	↑ ZO-1 expression and localization	Not determined	[287]
MOS (Shanghai Lanpu Bio. Co. Ltd.), MOS ≥ 20%, DP 2–9	0.6 g/kg BW/day	ETEC-exposed weaned piglets	In vivo	↑ ZO-1, claudin-1 mRNA	MD	[280]
MOS (Safmannan, Phileo Lesaffre Animal Care, Marcq-en-Baroeul, France)	0.5 g/kg BW/day	ETEC-exposed broilers	In vivo	↑ occludin mRNA, no effect in ZO-1, claudin-1 mRNA	MID	[288]
MOS (Safmannan, Phileo Lesaffre Animal Care, Marcq-en-Baroeul, France)	250 mg/kg BW/day	Heat stress-exposed broilers	In vivo	↑ occludin mRNA (jejunum), ↑ occludin, ZO-1 mRNA (ileum)	Not determined	[286]

DSS, Dextran sulfate sodium; ETEC, Enteropathogenic *E. coli*; FITC-D, Fluorescein isothiocyanate–dextran; KMOS, Konjac MOS; MD, Microbiota-dependent; MID, Microbiota-independent; MOS, Mannan oligosaccharides; PP, Paracellular permeability; TEER, Transepithelial electrical resistance; TJ, Tight junction.

### 4.6. Xylo-Oligosaccharides

#### 4.6.1. Structure and Sources

**Figure 8:**
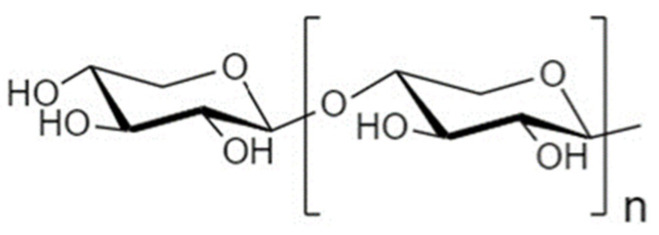
XOS structure: the main components of XOS are n 1,4-linked β-d-xylose monomers. XOS, xylooligosaccharides.

Xylooligosaccharides (XOS) are also characterized as novel health-beneficial oligosaccharides possessing prebiotic, immunomodulatory, antioxidant, antimicrobial, and anti-tumor properties [24,289]. XOS are linear oligomers composed of xylose units linked via β-(1→4) xylosidic bonds (Figure 8). The DP ranges between 2 and 7, forming xylo -biose, -triose, -tetraose, -pentaose, -hexaose, and -heptaose [290,291,292]. XOS can be naturally found in fruits, vegetables, milk, and honey in limited quantities [24,293]. For that reason, they are majorly produced by the chemical, physical or enzymatic degradation of xylan, a hemicellulose abundantly present in the plant cell wall [24,292].

#### 4.6.2. Microbiota-Dependent and Independent Effects on TJs

Based on in vitro [150,194] and in vivo studies on healthy [290,294] and LPS-challenged weaned piglets [295] and HFD-fed rats [296], XOS demonstrate barrier-protective and reinforcing properties related to their ability to modulate the gut flora (Table 6). By contrast, based on a study on healthy rats, even though XOS supplementation promotes the growth of Bifidobacteria and Lactobacilli, a limited effect on intestinal permeability is observed, probably due to insufficient gut flora alterations [291]. According to these studies, XOS enhance the growth of health-promoting bacteria, e.g., of the Bacteroidetes phylum, and reduce pro-inflammatory bacterial populations such as Proteobacteria, with parallel increases of butyrate, acetate, propionate, and valerate contents, and suppression of inflammatory activity. Treatment of DSS-challenged mice with XOS alone, or combined with the probiotic *B. infantis*, increases TJ gene expression by reducing the NLR family pyrin domain containing 3 (NLRP3) inflammasome mRNA levels, halting the secretion of caspase-1 and IL-1β [293]. In addition, XOS supplementation of broilers with XOS, or with a combination of XOS and the immunomodulatory polysaccharide γ-irradiated *Astragalus* polysaccharide (IAPS), seals the paracellular route through upregulation of TJ mRNA levels [297]. In both cases, XOS intake, combined with either the probiotic or IAPS, exerts a more potent effect, indicating synergistic underlying mechanisms. Nonetheless, the possibility of a correlation between gut flora alterations and the observed effects is not investigated in any of these two studies [293,297]. Not surprisingly, the closely related arabinoxylo-oligosaccharides also present barrier-protective activity in in vitro fermentation models [298,299].

A study on antibiotics-treated non-obese diabetic (NOD) mice suggests that XOS can also promote TJs independently of their prebiotic activity [300]. Indeed, XOS supplementation of the germ-free mice exerts direct effects on pancreatic islet pathology, which are accompanied by increased ZO-1 and occludin mRNA levels compared to the antibiotics-treated control group, but this effect is gut section-specific [300] (Table 6). Until now, the XOS-mediated microbiota-independent effects on IECs have been poorly investigated, and further in vitro and in vivo studies in germ-free settings are warranted.

## 5. Discussion

This review provides an overview of the currently available data regarding the regulation of intestinal epithelial TJs by various prebiotic NDOs. Preservation of strictly regulated and functional TJs is pivotal not only for gut homeostasis but also for an individual’s overall health. This is no surprise, considering their localization at the apicolateral borders of the IECs, rendering them the “gate-keepers” of the paracellular route. Besides the essential contribution to the formation of a semipermeable barrier to ions and solutes, TJs are vital for the conservation of cell polarity and signal transmission due to their role as signaling platforms. Compromised intestinal barrier function has been proposed as the origin and consequence of numerous intestinal and systemic disease states, though a clear separation of cause and effect in interpreting the significance of barrier loss is quite a challenge [8]. The occurrence of leaky gut syndrome signifies disrupted epithelial TJs, a state which is accompanied by increased translocation of commensals and pathogens into the body, systemic inflammation, and ultimately perturbation of the immune system homeostasis. Leaky gut-associated conditions include IBS, CD, UC, celiac disease, and intestinal graft versus host disease. However, numerous extra-intestinal diseases and disorders are also correlated with the leaky gut syndrome, such as NAFLD, obesity, metabolic syndrome, alcoholic cirrhosis, pancreatitis, asthma, eosinophilic esophagitis, Parkinson’s disease, autism, and stress-associated states [5].

Disturbed gut microbiota composition is closely related to elevated epithelial permeability due to a decrease in barrier-promoting microbes and an increase in inflammogenic ones. Even though probiotics are very promising with respect to maintaining intestinal barrier homeostasis and repairing lesions, there are potential drawbacks and limitations related to bacteriotherapy. Indeed, probiotic therapy in perinatal infants, immunocompromised individuals, and patients with impaired intestinal barrier function entails the risk of multisystemic infections caused by microbes, even the ones physiologically residing in the gut [12]. To date, no FDA-approved agents directly targeting the epithelial barrier are available [8]. Hence, uncovering agents that can specifically modulate TJ functionality and thus affect the leak, pore, and unrestricted pathways seems to be an appealing alternative to prevent or even treat conditions accompanied by increased epithelial permeability. Restrictions of probiotic therapy have brought prebiotics to the center of attention, promoting prebiotic supplementation and research. The NDOs have gained tremendous attention among the prebiotic agents due to their versatile properties, including anti-inflammatory, immunomodulatory, antitumor, antimicrobial, antioxidant, anti-apoptotic, neuroprotective, and anti-diabetic among others. In addition, ingestion of NDOs leads to a beneficial shift of the intestinal microbiota populations toward health-promoting microbes. The NDO-mediated restoration of dysbiosis alleviates or even abrogates leaky epithelia conditions via the positive regulation of TJs by the commensals. Modulation of TJ functionality occurs both directly, via bacterial cell structures, or indirectly via bacterial metabolites and excreted factors. Indeed, NDOs such as FOS, GOS, AOS, COS, MOS, and XOS discussed here, reinforce, and protect the epithelial barrier function via their prebiotic activities in both in vivo and in vitro models.

Apart from the microbiota-dependent barrier-protective effects of NDOs, emerging evidence demonstrates that these carbohydrate-based prebiotic fibers possess far more potential than that. ΝDOs seem to directly stimulate intestinal epithelial and immune cells, regulating inflammatory and immune responses to finally confer overall protection of the mucosal barrier [19,208]. Undeniable evidence supports that NDOs interact with cell surface receptors of IECs and stimulate various signaling pathways involved in TJ regulation, thus modulating paracellular permeability microbiota independently. An interesting uncovering of this study is that the extensively studied FOS and recently emerged COS share a common mechanism of TJ complex reinforcement: both oligosaccharides interact with the cell surface receptor CaSR, stimulating a CaSR-Gq-PLC-IP3-receptor channel-dependent cascade. As a result, there is a marked increase in CaMKKβ-mediated AMPK phosphorylation, which leads to acceleration of TJ re-assembly and thus the sealing of the paracellular route. GOS and MOS also seem to promote TJ assembly via the CaMKKβ/AMPK pathway and thus enhance TJ formation, though whether this occurs upon stimulation of CaSR, warrants further investigation. These findings seem to converge, indicating that NDOs can promote TJ functionality through AMPK signaling. Moreover, FOS, which are well-established TLR2 ligands in both immune and IECs, confer barrier protection via direct activation of TLR2/PKC/MAPK pathways involved in the regulation of TJ expression and physiological opening/sealing of the TJ pore. In addition, FOS and GOS strengthen the intact epithelial barrier without evident alterations in TJ abundance: the increased ionic resistance observed seems to be mediated by the induction of cell membrane-compositional changes and subsequent modifications in rigidity-fluidity, phenomena that eventually contribute to the formation of a robust epithelial layer. AOS also possess barrier-strengthening properties under homeostatic conditions. Upon binding to the MR, AOS induce TJ protein expression and decreased paracellular permeability. Considering that the MR also recognizes mannose and GlcNAc residues and that both MOS and COS reinforce the epithelial barrier under unchallenged conditions, their interaction with the MR is an appealing possibility that needs to be investigated. Regarding XOS, the available studies are mostly centered around the prebiotic-related effects on the epithelial barrier, though these NDOs also seem to exert direct effects on intestinal TJs, i.e., microbiota-independently.

**Figure 9:**
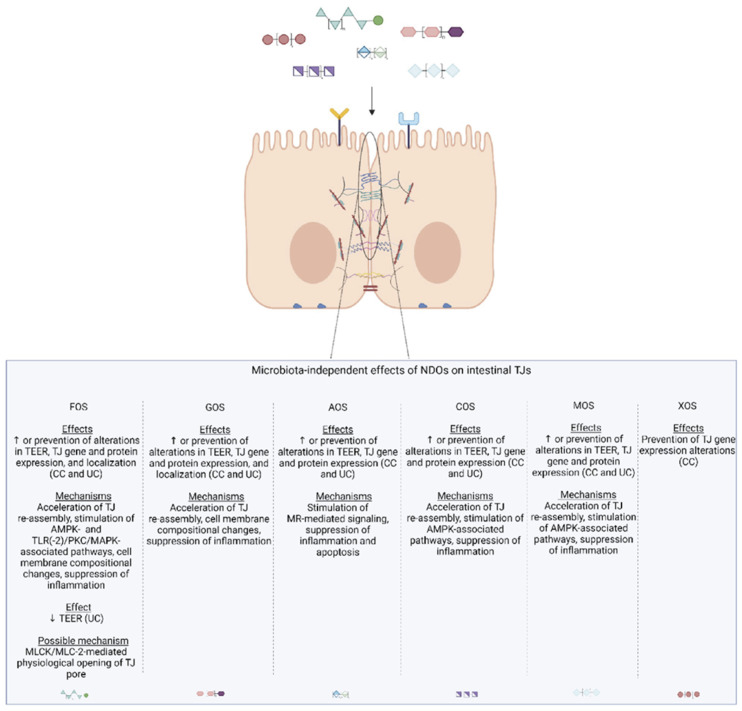
Synopsis of the NDO-mediated microbiota-independent effects on TJs: Apart from their prebiotic-associated barrier reinforcement, the NDOs interact with cell surface receptors including TLR, CaSR, and MR, positively modulating TJ functionality. NDOs can strengthen and protect the epithelial barrier integrity under normal and pathological conditions, preventing alterations in TJ expression and localization induced by injurious stimuli. These effects can be mediated by stimulation of TJ-regulatory effectors such as AMPK, PKC, and MAPK or by suppressing inflammatory and apoptotic pathways upon direct regulation of IECs and immune cells. CC: challenged conditions: treatment with NDO + a trigger; UC: unchallenged conditions: treatment with NDO solely. Created with BioRender.com. AMPK, Adenosine monophosphate-activated protein kinase; CaSR, Calcium-sensing receptor; MAPK, Mitogen-activated protein kinases; MR, Mannose receptor; NDO, Non-digestible oligosaccharide; PKC, Protein kinase C; TJ, Tight junctions; TLR, Toll-like receptor.

Finally, intestinal inflammation is a key contributor to barrier damage due to the noxious effects of the cytokines released from IECs and immune cells on the intestinal TJs. The NDOs suppress cytokine storms induced by noxious stimuli, mainly via TLR4/NF-κΒ- and MAPK-associated pathways, and thus save the monolayer integrity. Considering the complexity of signaling pathways involved in the regulation of TJs, their substantial crosstalk, and the lack of in-depth mechanistic studies, a clear distinction of the underlying mechanisms by which these NDOs exert direct barrier-protective effects is difficult to be achieved. In addition, numerous variables have a tremendous impact on the outcomes observed and underlying signaling pathways: the experimental model (in vitro, in vivo, or ex vivo) and setup, the type of trigger causing TJ impairments, the physicochemical characteristics (as determined by factors such as MW, DP, DD, PD, PA, purity, and source) of the NDOs mixtures, and concentrations/dosages of the treatments. Nonetheless, NDOs are undisputedly potent intestinal epithelial TJ complex regulators, both via their prebiotic activity and upon direct interactions with IECs. It is now well-established that NDOs modulate immune system activity via interactions with the lamina propria, possess anti-apoptotic properties, regulate Goblet cells for mucus production, and induce differentiation and epithelial wound repair, among others [19]. Hence, NDOs can modulate every component building up the intestinal barrier, ultimately conferring overall protection against external pathogens and contributing to homeostasis preservation. A synopsis of the key points regarding the microbiota-independent effects of NDOs on intestinal TJs is presented in Figure 9. Overall, based on the studies stated here, the potential beneficial health effects of prebiotics such as NDOs can be certainly acknowledged. However, considering that most of the available studies use in vitro and in vivo animal models, the shortcoming of the current knowledge is evident. Thus, the present extensive mechanistic knowledge should be leveraged to design rigorous human studies, first, to develop “personalized nutrition-based therapeutics” by correlating chronic inflammatory disorders with an optimal subset of patients within the disorder and testing the effectiveness of NDOs. Also, a second goal would be the establishment of “targeted nutrition-based therapeutics”, by identifying the most appropriate NDO (mixture) for a given disease and population. Finally, considering that NDOs are Generally Recognized as Safe (GRAS) nutritional supplements, the time lag between positive clinical results and release to the market will be short, highlighting their beneficial use over traditional pharmaceuticals.

## 6. Conclusions

The NDOs and putatively associated structures may have great potential against a leaky gut as potent intestinal epithelial TJ regulators via their microbiota-dependent and -independent modulation of TJ abundance and dynamics. 

## Figures and Tables

**Table 6:** Microbiota-dependent and independent effects of XOS and AXOS on paracellular permeability and/or TJs.

Treatment Characteristics	[(A)XOS]	Model/Experimental Setup	Type of Study	Observed Effects on PP and/or TJs	Type of Effect	References
AXOS (produced upon addition of xylanase to wheat bran)	10, 50 mg EP/kg (xylanase)	LPS-exposed Caco-2 monolayers incubated with AXOS ferment supernatant (using cecal content of broilers)	In vitro	↑ TEER	MD	[298]
AXOS-rich extract (Cosucra Groupe Warcoing S.A., Pecq, Belgium), 88% dietary fiber of which 66% AXOS, DS 0.38, DP 6	Fermentation of 5g/L AXOS	Caco2:THP-1 co-cultures incubated with AXOS ferment supernatant (using fecal batches of donors)	In vitro	↑ TEER	MD	[299]
XOS derived from corn, (AIDP, Inc., City of Industry, CA, USA), 70% XOS	0.5 g hydrated in 40 mL sterile trypticase peptone fermentation medium	Caco2:HT29-MTX co-cultures incubated with XOS ferment supernatant	In vitro	↑ TEER after 24 h on healthy gut model, no effect on TEER after 24 h on leaky gut model but ↓ LY flux	MD	[150]
XOS derived from sugarcane (Prenexus Health, Gilbert, AZ, USA), XOS 84%	3 g/day	SHIME^®^ inoculated with fecal sample from healthy donors and coupled with co-cultures of Caco2:HT29-MTX-E12	In vitro	↑ TEER	MD	[194]
XOS (Shandong Longlive, Qingdao, China)	ad libitum	NOD/MrkTac mice	In vivo	↓ FITC-Dextran flux	MD	[300]
				↑ ZO-1, occludin mRNA (only in the large intestine)	MID	
XOS alone or +*B. infantis*	0.23 g/kg BW/day	DSS-exposed mice (UC)	In vivo	↑ ZO-1, occludin, claudin-1 mRNA (better effect as synbiotic)	Not determined	[293]
XOS (Shandong Longlive Biotechnology Co., Ltd., Dezhou, China)	0.02% XOS containing 35% XOS with 65% maltodextrin as carrier	LPS-exposed weaned piglets	In vivo	↑ claudin-1 in both unchallenged and challenged animals	MD	[295]
XOS (Jiangsu Kangwei Biologic Co., Ltd., Dongtai, China)	0.01% XOS	Weaned piglets	In vivo	↑ ZO-1, tendency to ↑ occludin, no effect on claudin-1 abundance	MD	[294]
XOS (Shandong Longlive Bio-technology Co. Ltd., Shandong, China), containing xylobiose, xylotriose, and xylotetraose at ≥35%	100 g/t	Weaned piglets	In vivo	↑ claudin-2 mRNA (compared to positive control group	MD	[290]
	250 g/t			↑ ZO-1 mRNA, no effect on occludin, claudin 2,3 (compared to basal diet-fed animals		
XOS (Jiangsu Kangwei Biological Co., Ltd., Nanjing, China), 35% XOS + IAPS	100 mg/kg	Broilers	In vivo	↑ ZO-1, occludin, claudin-1, -3 mRNA (better effect with combined treatments)	Not determined	[297]
	100 mg/kg + 600 mg/kg IAPS					
XOS (Shandong Longlive Bio-Technology Co., Ltd., China), 95% pure powder extracted from corncob	500 mg/mL (2 mL every 2nd day)	Rats	In vivo	No effect on FITC-D permeability on plasma, nor on TEER (Caco-2 cells treated with cecal water), ↑ occludin mRNA	MD	[291]
XOS (Shandong Longli Biotechnology Co., Shandong, China), 95% XOS	1 g/kg BW/day	HFD-fed rats	In vivo	↑ occludin mRNA	MD	[296]

AXOS, Arabino-XOS; DSS, Dextran sulfate sodium; FITC-D, Fluorescein isothiocyanate–dextran; HFD, High fat diet; MD, Microbiota-dependent; MID, Microbiota-independent; PP, Paracellular permeability; SHIME, Simulator of the Human Intestinal Microbial Ecosystem; TEER, Transepithelial electrical resistance; TJ, Tight junction; XOS, Xylooligosaccharides.

## Data Availability

Not applicable.

## Abbreviations

[Ca^2+^]I	Intracellular Ca^2+^
*A. muciniphila*	*Akkermansia muciniphila*
AhR	Aryl hydrocarbon receptor
AJs	Adherens junctions
AmEVs	*A. muciniphila*-originated EVs
AMP	Antimicrobial peptide
AMPK	Adenosine monophosphate (AMP)-activated protein kinase
AOS	Alginate-oligosaccharides
*B. fragilis*	*Bacteroides fragilis*
BiCM	*B. infantis* conditioned medium
CaMKKβ	Ca^2+^/calmodulin-dependent kinase kinase β
CaSR	Calcium-sensing receptor
CD	Crohn’s disease
CDX2	Caudal type homeobox 2
CLR	C-type lectin-like receptor
COS	Chitin/chitosan-oligosaccharides
CRC	Colorectal cancer
DA	Degree of acetylation
DD	Degree of deacetylation
DON	Mycotoxin deoxynivalenol
DP	Degree of polymerization
DSS	Dextran sulfate sodium
*E. coli*	*Escherichia coli*
EcN	*Escherichia coli* Nissle 1917
EHEC	Enterohemorrhagic *E*. *coli*
ERK	Extracellular signal-regulated kinase
ETEC	Enteropathogenic *E. coli*
EVs	Extracellular vesicles
*F. prausnitzii*	*Faecalibacterium prausnitzii*
F-actin	Filamentous actin
FITC-D	Fluorescein isothiocyanate–dextran
FMT	Fecal microbiota transplantation
FOS	Fructooligosaccharides
GAPs	GTPase-activating proteins
GDIs	Guanine nucleotide dissociation inhibitors
GEFs	Guanine nucleotide exchange factors
GI	Gastrointestinal
GJs	Gap junctions
GlcNAc	*N*-acetyl-d-glucosamine
GOS	Galactooligosaccharides
HDAC	Histone deacetylase
HFD	High-fat diet
HMOs	Human milk oligosaccharides
HMW	High MW COS
IAPS	γ-irradiated *Astragalus* polysaccharide
IBD	Inflammatory bowel disease
IBS	Irritable bowel syndrome
ICAM-1	Intercellular adhesion molecule-1
IECs	Intestinal epithelial cells
IgA	Immunoglobulin A
IL	Interleukin
INF-γ	Interferon-γ
IP3	D-myo-inositol 1,4,5-trisphosphate
JAM	Junctional adhesion molecules
JNK	c-Jun N-terminal kinase
KMOS	Konjac MOS
LKB1	Liver Kinase B1
LMW	Low MW
LPS	Lipopolysaccharide
LY	Lucifer yellow
MAMPs	Microbial-associated molecular patterns
MAPK	Mitogen-activated protein kinase
MCD	Methionine-choline-deficient
MD	Microbiota-dependent
MID	Microbiota-independent
MLC-2	Myosin II regulatory light chain
MLCK	Myosin light chain kinase
MOS	Mannan-oligosaccharides
MR	Mannose receptor
mTOR	Mammalian target of rapamycin
MW	Molecular weight
NAFLD	Non-alcoholic fatty liver disease
NASH	Non-alcoholic steatohepatitis
NDOs	Non-digestible oligosaccharides
NM 2	Non-muscle myosin 2
NOD	Non-obese diabetic
Nrf2	Nuclear factor erythroid 2-related factor 2
OMVs	Outer membrane vesicles
PD	Polydispersity
PDZ	PSD95–DlgA–ZO-1 homology
PI3K	Phosphatidyl inositol-3 kinase/phosphoinositide 3-kinases
PKC	Protein kinase C
PLC	Phospholipase C
PMA	Phorbol 12-myristate 13-acetate
POS	Pectic-oligosaccharides
PP	Paracellular permeability
PP	Protein phosphatase
PRRs	Pattern recognition receptors
ROCK	Rho-associated coiled coil containing protein kinase
SAP	Severe acute pancreatitis
SCFAs	Short-chain fatty acids
SHIME	Simulator of the Human Intestinal Microbial Ecosystem
SIGNR1	Specific intercellular adhesion molecule-3-grabbing nonintegrin-related 1
SLPs	Surface layer proteins
SOS	Soybean oligosaccharides
TAMPS	TJ-associated MARVEL proteins
TEER	Transepithelial electrical resistance
TGF-β	Transforming growth factor-β
TJ	Tight junction
TLR	Toll-like receptor
UAOS	Unsaturated AOS
UC	Ulcerative colitis
UroA	Urolithin A
XOS	Xylooligosaccharides
ZO	Zonula occludens
